# Ion‐Scale Magnetic Flux Rope Generated From Electron‐Scale Magnetopause Current Sheet: Magnetospheric Multiscale Observations

**DOI:** 10.1029/2022JA031092

**Published:** 2023-03-24

**Authors:** H. Hasegawa, R. E. Denton, K. Dokgo, K.‐J. Hwang, T. K. M. Nakamura, J. L. Burch

**Affiliations:** ^1^ Institute of Space and Astronautical Science Japan Aerospace Exploration Agency Sagamihara Japan; ^2^ Southwest Research Institute San Antonio TX USA; ^3^ Department of Physics and Astronomy Dartmouth College Hanover NH USA; ^4^ Space Research Institute Austrian Academy of Sciences Graz Austria

**Keywords:** magnetic reconnection, magnetopause, magnetic flux rope, flux transfer event, energy conversion

## Abstract

We present in‐depth analysis of three southward‐moving meso‐scale (ion‐to magnetohydrodynamic‐scale) flux transfer events (FTEs) and subsequent crossing of a reconnecting magnetopause current sheet (MPCS), which were observed on 8 December 2015 by the Magnetospheric Multiscale spacecraft in the subsolar region under southward and duskward magnetosheath magnetic field conditions. We aim to understand the generation mechanism of ion‐scale magnetic flux ropes (ISFRs) and to reveal causal relationship among magnetic field structures, electromagnetic energy conversion, and kinetic processes in magnetic reconnection layers. Results from magnetic field reconstruction methods are consistent with a flux rope with a length of about one ion inertial length growing from an electron‐scale current sheet (ECS) in the MPCS, supporting the idea that ISFRs can be generated through secondary reconnection in an ECS. Grad‐Shafranov reconstruction applied to the three FTEs shows that the FTEs had axial orientations similar to that of the ISFR. This suggests that these FTEs also formed through the same secondary reconnection process, rather than multiple X‐line reconnection at spatially separated locations. Four‐spacecraft observations of electron pitch‐angle distributions and energy conversion rate $j\cdot E^{\prime }=j\cdot \left(E+v_{e}\times B\right)$ suggest that the ISFR had three‐dimensional magnetic topology and secondary reconnection was patchy or bursty. Previously reported positive and negative values of $j\cdot E^{\prime }$, with magnitudes much larger than expected for typical MP reconnection, were seen in both magnetosheath and magnetospheric separatrix regions of the ISFR. Many of them coexisted with bi‐directional electron beams and intense electric field fluctuations around the electron gyrofrequency, consistent with their origin in separatrix activities.

## Key Points

- Ion‐scale magnetic flux rope (ISFR) can be generated from reconnecting electron‐scale current sheet at the subsolar magnetopause (MP)
- Preceding mesoscale flux ropes had axial directions akin to that of the ISFR in the MP, suggesting the same generation mechanism
- The ISFR had complex magnetic topology with three‐dimensional effects and involved patchy, intense energy conversion in separatrix regions

## 1 Introduction

Magnetopause (MP) reconnection often occurs in a time‐dependent and/or localized manner, generating flux transfer events (FTEs). FTEs are characterized by a bipolar variation of the magnetic field component normal to the nominal MP and also generally by an enhancement of the field magnitude around the event center (see Raeder (2006) and Hasegawa (2012) for reviews). While large‐scale FTEs (typically with dimensions ∼1$R_{E}$) have been extensively investigated both theoretically and observationally and their formation processes are relatively well known (e.g., Fear et al., 2012), little is known about the generation mechanism of ion‐scale FTEs or magnetic flux ropes (FRs) with sizes comparable to or somewhat larger than ion inertial length (typically ∼50 km at the dayside MP). They have been observed in exhaust regions of both MP (Eastwood et al., 2016) and magnetotail (Stawarz et al., 2018) reconnection.

Numerical simulations of magnetic reconnection show that ion‐scale FRs can form through secondary magnetic reconnection in an elongated portion of reconnecting current sheets with electron‐scale thicknesses. Two‐dimensional kinetic simulations show that secondary reconnection can occur regardless of the presence (Drake et al., 2006) or absence (Daughton et al., 2006; Hesse et al., 1999) of guide field, the magnetic field component in the direction of X‐line. Dong et al. (2017) reported observations by the Magnetospheric Multiscale (MMS) spacecraft (Burch et al., 2016) of three consecutive FTEs, which were followed by an encounter of reconnecting electron‐scale current sheet (ECS) (Burch & Phan, 2016), and suggested that these FTEs formed through secondary reconnection in the ECS. Recently, Hasegawa et al. (2022) demonstrated that electron‐scale magnetic islands (FRs in the absence of guide field) can form and grow in an ECS of magnetotail reconnection. Although these observations (Dong et al., 2017; Eastwood et al., 2016; Hasegawa et al., 2022) are consistent with the secondary reconnection scenario, there is a gap in our understanding about the link between electron‐scale FRs in reconnecting ECS and ion‐scale FRs in large‐scale reconnection jets or exhausts; can electron‐scale FRs grow to form ion‐scale FRs? This question needs attention because electron‐scale FRs may be dissipated in reconnecting ECS through magnetic field annihilation, which was recently confirmed in simulations by Nakamura et al. (2021) and observations by Hasegawa et al. (2022).

Another interesting feature of ECSs observed at the MP is that the energy conversion rate $j\cdot E^{\prime }=j\cdot \left(E+v_{e}\times B\right)$ (Zenitani et al., 2011) in or around MP electron diffusion regions (EDRs) often exceeds the values ($\leq 4\;nW/m^{3}$) expected for typical MP reconnection (Webster et al., 2018). What is peculiar is that $j\cdot E^{\prime }$ often exhibits oscillatory or bipolar features, with both positive and negative values (Burch et al., 2018; Genestreti et al., 2017). Genestreti et al. (2022) discussed possible causes of these intense and oscillatory energy conversion signatures and effects of upstream (magnetosheath) and boundary conditions. However, since the reconnection region geometry and spacecraft path with respect to reconnection layer structures are often hardly known from in‐situ measurements, their origins are not fully understood.

In the present study, we revisit MMS observations of MP FTEs and ECS on 8 December 2015, reported by Dong et al. (2017) and Burch and Phan (2016), respectively. The ECS in this event showed multiple oscillatory $j\cdot E^{\prime }$ features (Burch et al., 2018), and thus the event is ideal for investigating both the FTE generation process and origin of anomalous energy conversion in ECS. The structures of the FTEs and MP are recovered by Grad‐Shafranov reconstruction (GSR) and electron magnetohydrodynamics (EMHD) reconstruction methods, respectively. Our intents are to reveal not only the generation mechanism of ion‐scale FRs but also the interrelationship among the magnetic field structure, oscillatory $j\cdot E^{\prime }$, and kinetic processes responsible for energy conversion in the ECS.

The outline of the present paper is as follows. In Section 2, an overview is given of MMS observations surrounding the MP current sheet (MPCS) on 8 December 2015. In Section 3, results from both the GSR and EMHD reconstruction are presented and discussed. In Section 4, detailed discussions are given about three‐dimensional aspects of the MPCS, origins of the oscillatory $j\cdot E^{\prime }$, and formation and decay processes of FTEs. A brief summary is presented in Section 5.

## 2 Event Overview and Data

**Figure 1:**
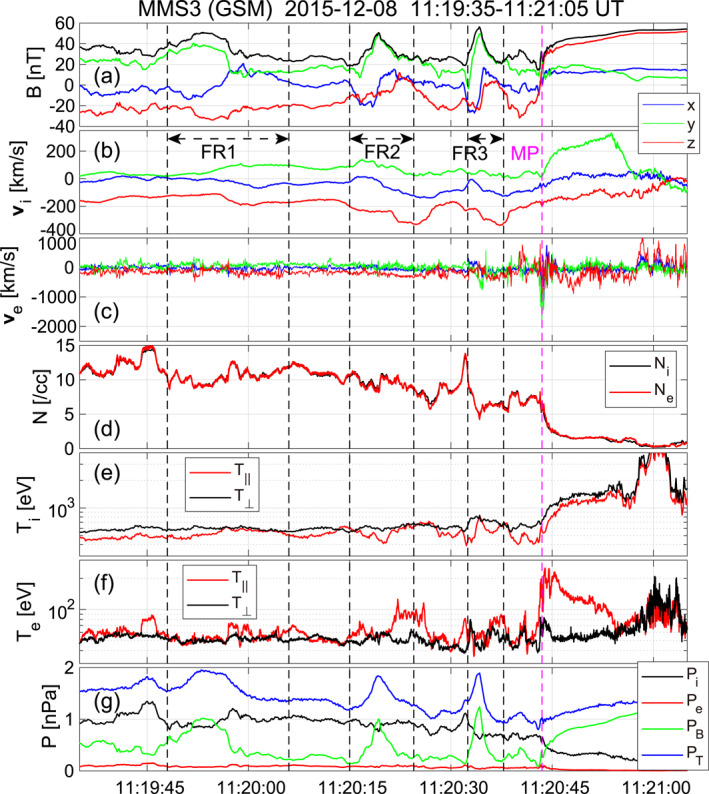
Burst‐mode data during three flux transfer events (FTEs) and magnetopause (MP) current sheet crossing recorded by the MMS3 spacecraft on 8 December 2015. Three FTEs were interpreted as being magnetic flux ropes (FRs) by Dong et al. (2017) and are called as such in the present paper. (a) Magnetic field in geocentric solar magnetospheric (GSM) coordinates, (b) ion velocity at 150 ms resolution, (c) electron velocity at 30 ms resolution, (d) ion and electron densities, (e) ion temperatures in the directions parallel and perpendicular to the magnetic field, (f) parallel and perpendicular temperatures of electrons, and (g) ion, electron, magnetic, and total pressures. Three sets of vertical black dashed lines indicate the time intervals used for the Grad‐Shafranov reconstruction. All figures except for Figure 8 of the present paper were created by Matlab (see Data Availability Statement of Hasegawa et al. (2021)).

**Figure 2:**
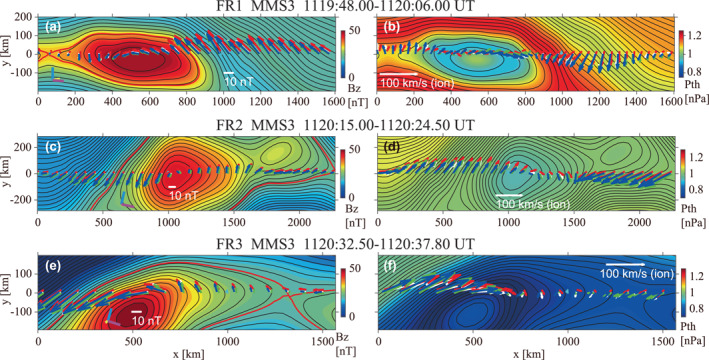
Two‐dimensional (2D) magnetic fields and pressures for the three flux transfer events (FTE)s (FR1, FR2, and FR3), reconstructed from MMS3 data by use of the Grad‐Shafranov reconstruction (GSR) method. Black curves show the reconstructed in‐plane magnetic field lines, with the axial component $B_{z}$ in color in panels (a, c, e) and plasma thermal pressure in color in panels (b, d, f). The white, red, green, and blue arrows in panels (a, c, e) are the projections onto the reconstruction (*x*–*y*) plane of the magnetic fields measured by MMS1, MMS2, MMS3, and MMS4, respectively, and those in panels (b, d, f) are the measured ion velocities transformed into the deHoffmann‐Teller frame. The cyan, yellow, and magenta bars near the lower‐left corner of panels (a, c, e) are the projections of the unit vectors of the geocentric solar magnetospheric (GSM) *x*, *y*, and *z* axes, respectively. The in‐plane flux contents inside the FTEs bounded by the red curve in (a, c, and e) are shown in Table 1.

Figure 1 shows an overview of observations by the MMS3 spacecraft near the subsolar MP of three FTEs, reported by Dong et al. (2017), and an ECS, reported by Burch and Phan (2016) and Burch et al. (2018), on 8 December 2015, 11:19:35–11:21:05 UT. MMS was located at (10.2, 1.3, −1.4) $R_{E}$ in GSM. Since near the subsolar point the normal direction of the nominal MP is roughly along the $x_{GSM}$ axis, $B_{x}$ can be taken as the normal field component. For each of the three FTEs, negative to positive $B_{x}$ variation as a typical signature of southward moving FTEs is clearly seen during the interval sandwiched between the dashed black vertical lines (Figure 1a). Consistently, southward reconnection ion jets were observed, in particular, during trailing parts of the FR2 and FR3 intervals (Figure 1b). A density enhancement, likely caused by compression on the leading side of FTEs, was observed immediately before FR3 (Figure 2d). In the present event, the static pressure in the magnetosheath (observed during an earlier part of the interval) was dominated by ion thermal pressure. A peak in the static pressure as another typical signature of FTEs was clearly seen at or near the center of all three FTEs (Figure 1g). The pressure increase was due to an enhancement of the core magnetic field component, which was roughly $B_{y}$ in the present case (Figure 1a).

Following these three FTEs, MMS crossed a MPCS at 11:20:43 UT, as indicated by high‐speed electron flows predominantly in the $-y_{GSM}$ direction (Figure 1c), from the magnetosheath into the magnetosphere. The electron temperature strongly increased during this crossing, especially in the direction parallel to the magnetic field (Figure 1f). Possible electron heating processes are discussed in Section 4.2. This current sheet was interpreted to be of an electron diffusion region (EDR) by Burch and Phan (2016), but we will show in Sections 3.2 and 4 that it involved complex magnetic structures and energy conversion processes not close to X‐line(s) but in separatrix regions.

MMS data used in the present study are magnetic fields from the fluxgate magnetometers (Russell et al., 2016), electric fields from the double‐probe instruments (Ergun, Goodrich, et al., 2016; Ergun, Tucker, et al., 2016; Lindqvist et al., 2016), and ion and electron moments and electron velocity distributions from the Fast Plasma Investigation instrument suite (Pollock et al., 2016).

## 3 Reconstruction of FTEs and MPCS

In order to gain insights into the generation process of the observed FTEs, we reconstruct magnetic structures of the three FTEs and the MPCS from MMS data. GSR for recovering two‐dimensional (2D) magnetohydrostatic structures (Hau & Sonnerup, 1999; Sonnerup et al., 2006) is applied to the three FTEs. A method for recovering 2D sub‐ion‐scale reconnection regions, known as the EMHD reconstruction (Hasegawa et al., 2021; Sonnerup et al., 2016), and polynomial reconstruction technique for three‐dimensional (3D) magnetic field structures (Denton et al., 2020, 2022; Torbert et al., 2020) are used to analyze the MPCS.

### 3.1 Grad‐Shafranov Reconstruction

GSR is a single‐spacecraft method for the reconstruction of 2D magnetic field structures on the MHD scale from magnetic field and plasma pressure data. It allows for the production of 2D field maps in a region around the path of an observing spacecraft, and an estimation of the FR orientation or invariant axis ${\hat{z}}_{GSR}$ along which spatial gradients are assumed to be negligibly small (Hu & Sonnerup, 2002). In addition to the 2D assumption, the magnetohydrostatic force balance is assumed; inertia terms in the MHD momentum equation can be neglected and thus the force from gradient of the total static pressure is approximately balanced with magnetic tension. The reconstruction is performed in a moving frame in which the structure looks approximately stationary, which is the deHoffmann‐Teller (HT) frame (Khrabrov & Sonnerup, 1998) in the GSR case. The reconstruction coordinate system is defined as follows: ${\hat{x}}_{GSR}$ is antiparallel to the projection of the HT velocity $V_{HT}$ onto the plane perpendicular to ${\hat{z}}_{GSR}$, and ${\hat{y}}_{GSR}={\hat{z}}_{GSR}\times {\hat{x}}_{GSR}$ forms the right‐handed orthogonal system. In this coordinate system, the spacecraft is seen to move in time along the ${\hat{x}}_{GSR}$ axis in the reconstruction plane and at an axial velocity $-V_{HT}\cdot {\hat{z}}_{GSR}$.

Figure 2 shows 2D maps of the magnetic field and plasma pressure from the GSR method applied to MMS3 data for the three FTEs. The spacecraft separation ∼15 km was small on the MHD scale, so that the use of different spacecraft makes no significant difference. Table 1 shows parameters used in or obtained from the reconstruction. The HT analysis was applied to combined ion velocity and magnetic field data with 150 ms resolution. For all three FTEs, the HT velocity is dominantly southward, with a duskward component. This is consistent with southward motion of the FTEs and the direction of reconnection jets expected on the southern side of dayside X‐line under continuously southward and duskward magnetosheath field conditions (Figure 1a). The Walén relation (Paschmann & Sonnerup, 2008) is weakly satisfied, with its slope magnitude not close to unity; inertia effects were only modestly important in the MHD force balance. On the other hand, the negative slopes indicate that the three FTEs were encountered in or near reconnection exhausts on the southern side of the X‐line(s) that generated these FTEs.

**Table 1:** Results Summarizing the Grad‐Shafranov Reconstruction Applied to Three Flux Transfer Events

	FR1	FR2	FR3
Time interval (UT)	11:19:48.0–11:20:06.0	11:20:15.0–11:20:24.5	11:20:32.5–11:20:37.8
$V_{HT}$ ^a^ (km/s in GSM)	(–23.5, 112.3, −178.7)	(–39.2, 195.2, −265.0)	(–75.5, 101.4, −283.8)
Walen slope^b^	−0.234	−0.454	−0.205
Invariant axis $\hat{z}$ (GSM)	(–0.1245, 0.8352, −0.5356)	(0.0559, 0.9884, −0.1412)	(0.1634, 0.9754, 0.1477)
$\hat{x}$ (GSM)	(–0.0052, 0.5393, 0.8421)	(0.2163, 0.1261, 0.9682)	(0.2693, −0.1881, 0.9445)
$\hat{y}$ (GSM)	(0.9922, 0.1077, −0.0628)	(0.9747, −0.0846, −0.2067)	(0.9491, −0.1146, −0.2934)
In‐plane flux content (Tesla·meter)	$2.0\times 10^{-3}$	$6.5\times 10^{-3}$	$6.0\times 10^{-3}$
CC_B_ ^c^	0.9967	0.9966	0.9941
*θ* ^d^ (degree)	59.0	32.7	15.3

^a^ deHoffmann‐Teller (HT) velocity (Khrabrov & Sonnerup, 1998).

^b^ Slope of the regression line in a scatter plot of the components of ion velocities measured in the HT frame and those of the local Alfvén velocities during the interval (Paschmann & Sonnerup, 2008).

^c^ The correlation coefficient between the GSM components of the magnetic field measured by, and those predicted from the GSR map along the paths of, the three MMS spacecraft not used in the reconstruction.

^d^ Angle between the flux rope axis and that of the ion‐scale flux rope in the magnetopause current sheet observed at 1120:43 UT (Figure 4).

For all three FTEs, the field maps show a FR structure with an intense core field component $B_{zGSR}$ comparable to 50 nT. The FR lengths were roughly 1,000 km, which was ∼14$d_{i}$ with ion inertial length in the magnetosheath $d_{i}∼70$ km; all three FTEs were of MHD scale, but much smaller than typical FTEs with sizes ∼1$R_{E}$ (Hasegawa et al., 2006, 2010). The invariant axis was roughly oriented in the $y_{GSM}$ direction, which was roughly the X‐line orientation expected for the present external field conditions.

**Figure 3:**
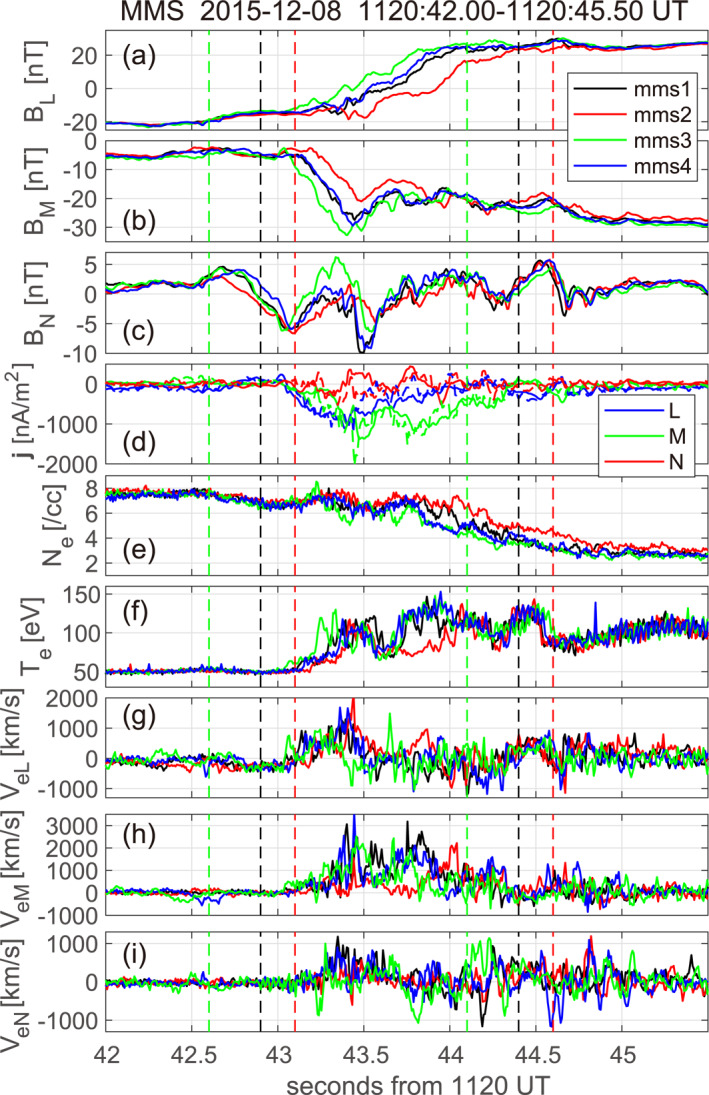
Data at 7.5 ms resolution from the four MMS spacecraft for the magnetopause current sheet at 11:20 UT. (a–c) LMN components of the magnetic field, (**d**) current density at the MMS centroid from particle measurements (solid) and $\left(\nabla \times B\right)/\mu_{0}$ (dashed), (**e**) electron density, (**f**) electron temperature assuming isotropy, and (**g‐i**) electron velocity. The LMN axes are estimated from optimization of the electron MHD (EMHD) reconstruction results (see text for details). The intervals sandwiched between pairs of black, red, green, and blue vertical dashed lines are used for the EMHD reconstruction from MMS1, MMS2, MMS3, and MMS4 data, respectively (the intervals for MMS1 and MMS4 are the same and thus the blue lines are not seen).

The in‐plane flux content, defined as the amount of in‐plane magnetic flux embedded inside the red field lines in Figures 2a, 2c, and 2e is of order $5\times 10^{-3}$ Tesla meter. This is roughly one order of magnitude smaller than the flux content for typical FTEs (Hasegawa et al., 2006). For the Alfvén speed ∼150 km/s based on the reconnecting magnetic field component ∼20 nT and density ∼8 ${cm}^{-3}$ (Figures 3a and 3e), the reconnection electric field expected for the dimensionless reconnection rate 0.1–0.2 (Liu et al., 2017) is 0.3–0.6 mV/m. Assuming that this reconnection electric field was sustained during the present event, the in‐plane flux reconnected per 20 s, which was roughly the interval between two neighboring FTEs in the present event (Figure 1), is $6\times 10^{-3}$ to $1.2\times 10^{-2}$ Tesla meter. This value is comparable to the flux content of the three FTEs (Table 1), which suggests that magnetic reconnection was continuously active at the X‐line that produced the FTEs and the corresponding reconnection region kept generating FTEs.

### 3.2 EMHD Reconstruction of the MPCS

The EMHD reconstruction is a single‐spacecraft method for the reconstruction of 2D electromagnetic and electron velocity fields in and around the EDRs of magnetic reconnection from magnetic field, electric field, and electron moment data (Sonnerup et al., 2016). It is based on a 2D and time‐independent form of the electron MHD equations assuming isotropy for the diagonal components of the electron pressure tensor. The most recent version allows for incorporating the effects of electron inertia, nonuniform electron density and temperature, and guide magnetic field (Hasegawa et al., 2021). A first trial reconstruction is usually performed in a structure‐rest frame estimated by the Spatio‐Temporal Difference (STD) method (Shi et al., 2019) and using an invariant axis (${\hat{z}}_{EMHD}$) orientation or M direction estimated by a hybrid method (Denton et al., 2018). The hybrid method is based on combined use of the minimum variance analysis of the magnetic field and Maximum Directional Derivative method (Shi et al., 2019) applied to four‐spacecraft measurements of the magnetic field (MDDB). EMHD reconstruction coordinates are defined in a similar manner to the GSR method. The final frame velocity $V_{str}$ and coordinate system used for optimized reconstruction results are determined by a trial‐and‐error method (Hasegawa et al., 2017). It attempts to maximize the correlation coefficient between the normalized components of the measured magnetic field and electron velocity and those predicted from the reconstructed field maps along the paths of the three spacecraft not used as input for the reconstruction.

For the EMHD reconstruction, we use magnetic field, electric field, and electron moment data at 7.5 ms resolution (Rager et al., 2018), rather than 30 ms resolution of level‐2 burst mode. The aim is to reveal fine scale structures in the current sheet of energy conversion and electron temperature which may have implications for kinetic and electron energization processes underlying energy conversion. Figure 3 shows the data from all four MMS spacecraft for 11:20:42.0–11:20:45.5 UT surrounding the reconstructed intervals. LMN coordinates in Figure 3 are the final ones used in the reconstruction: $L_{EMHD}=\left(0.3089,-0.4365,0.8468\right)$, $M_{EMHD}=\left(-0.1692,-0.8995,-0.4029\right)$, and $N_{EMHD}=\left(0.9376,-0.0209,-0.3472\right)$ in GSM.

**Figure 4:**
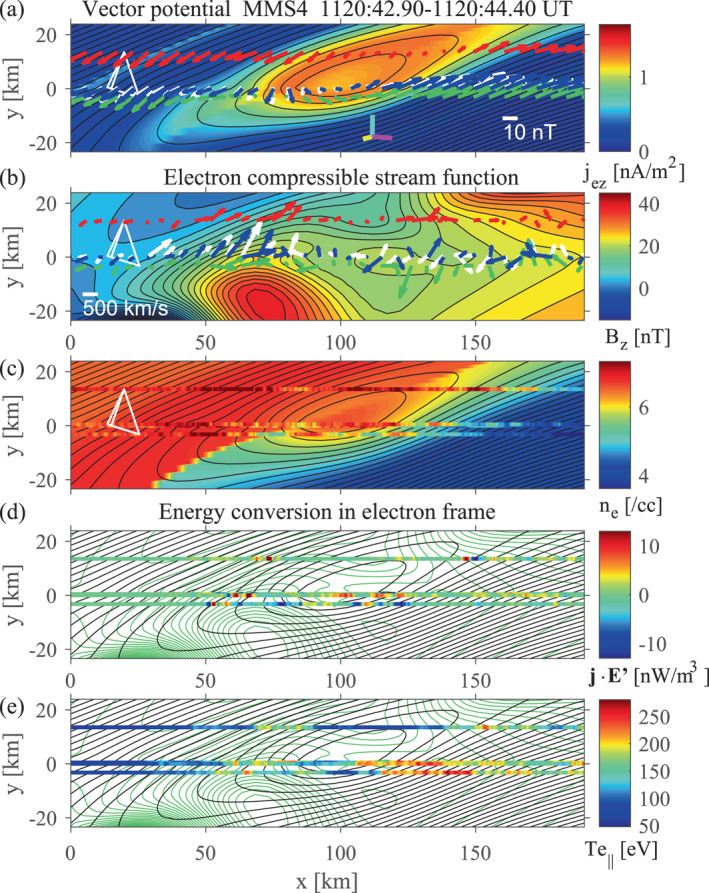
Field maps from the electron magnetohydrodynamics (EMHD) reconstruction method with electron inertia and compressibility effects (Hasegawa et al., 2021) applied to MMS4 data with 7.5 ms resolution. (a) Reconstructed in‐plane magnetic field, with the recovered out‐of‐plane current density in color. (b) Electron streamlines predicted from the reconstructed out‐of‐plane magnetic field component in color. (c) Reconstructed electron density, with electron densities measured along the paths of the four spacecraft in color. (d and e) In‐plane field lines (black) and electron streamlines (green), with measured electron‐frame energy conversion rates $j\cdot E^{\prime }=j\cdot \left(E+v_{e}\times B\right)$ (Zenitani et al., 2011) (d) and parallel electron temperatures (e) in color. The white, red, green, and blue arrows in (a) are the projections onto the reconstruction (*x*–*y*) plane of the magnetic fields measured by MMS1, MMS2, MMS3, and MMS4, respectively, while those (b) are the measured electron velocities in the structure‐rest frame. The cyan, yellow, and magenta bars in (a) are the projections of the unit vectors of the GSM *x*, *y*, and *z* axes, respectively. In (c–e), the MMS4 paths colored by the measured electron density, energy conversion rate, and electron temperature, respectively, are nearly on top of the MMS1 paths.

The current density was intense (Figure 3d) and comparable to those seen in EDRs reported in the literature (Burch et al., 2016; Webster et al., 2018). The magnitude of the electron velocity component $v_{eM}∼3,000$ km s^−1^ (Figure 3h) was about half the electron Alfvén speed $V_{eA}$ ∼ 6,400 km s^−1^, consistent with the presence of ECS in the MPCS. The $B_{N}$ component shows substantial variations (Figure 3c) and the current density component $j_{M}$ is not single‐peaked (Figure 3d), suggesting complex structures in the MPCS. However, the MDDB analysis shows that the intermediate eigenvalue (equivalent to squared magnetic gradient in local L) is much larger than the minimum eigenvalue (squared magnetic gradient in local M) around the MPCS center (Figure S1 in Supporting Information S1). This suggests that the local magnetic structure was approximately 2D, permitting the use of the EMHD method. On the other hand, the data variations in Figure 3 appear very different among the four spacecraft, in particular for $B_{M}$, $B_{N}$, and $v_{eM}$, during the crossing. Provided the fact that the spacecraft separation ∼15 km was comparable to the thickness of the MPCS (Figure 4a), this suggests that the four spacecraft saw different portions of the MPCS structure and/or different phases of MPCS evolution.

Figure 4 shows field maps recovered from the up‐to‐date version of the EMHD reconstruction using MMS4 data. The structure velocity is $V_{str}=\left(-7.0,15.7,-132.7\right)$ km s^−1^ in GSM, which is not extremely different from the average ion velocity during the interval $\langle V_{i}\rangle =\left(-42.8,38.9,-172.1\right)$ km s^−1^ (the L and N components of $V_{str}$ were optimized by the trial‐and‐error method, but its M component was taken to be that of $\langle V_{i}\rangle$). GSM components of the reconstruction axes are: ${\hat{x}}_{EMHD}=\left(-0.0012,-0.4086,0.9127\right)$, ${\hat{y}}_{EMHD}=\left(0.9856,-0.1549,-0.0681\right)$, and ${\hat{z}}_{EMHD}=\left(0.1692,0.8995,0.4029\right)=-M_{EMHD}$. The thickness of the reconstructed MPCS is consistent with that of the current sheet, 19.6 km (∼9$d_{e}$ with the electron inertial length $d_{e}=2.2$ km), estimated from the normal velocity, $V_{str}\cdot N_{EMHD}=39.2$ km s^−1^, and time scale ∼0.5 s of the crossing (Figure 3a). Interestingly, the MPCS contains a magnetic FR with a length ∼100 km, comparable to $d_{i}$ (Figure 4a). Thus, this FR is hereafter referred to as the ion‐scale flux rope (ISFR). It is possible that MMS4 saw a thickened (sub‐ion‐scale) portion of the MPCS in the presence, and/or as a result of the growth, of the FR. In Section 3.3, we argue that the ISFR likely formed from an ECS.

The out‐of‐plane field component $B_{z}$ has a peak not at the ISFR center but on the southern (lower‐left in the map) side of the ISFR center (Figure 4b). This might be associated with 3D effects, as will be discussed in Section 4.1. For each (magnetosheath or magnetospheric) side of the MPCS, the electron density and temperature are assumed to be preserved along the in‐plane field lines (Hasegawa et al., 2021). Thus, the reconstructed density shows abrupt jumps at the MPCS center on some field lines (Figure 4c), because separate branches of the function $n_{e}\left(A\right)$ are used on the two sides and the branch is switched by the polarity of $B_{L}$ (see Hu & Sonnerup (2003) for similar approach). Note also that under the model assumptions, $B_{z}$ (as shown in Figure 4b) is equivalent to the compressible electron stream function ψ via $B_{z}=-\mu_{0}e\psi$ (Hasegawa et al., 2021), so that surfaces of constant $B_{z}$ are parallel to the in‐plane electron velocity. This physically makes sense because in steady, 2D ion diffusion regions, $B_{z}$ (Hall magnetic field) is generated by the in‐plane components of the electron current $-en_{e}v_{e}$.

The reconnection electric field $E_{z0}$, which is optimized and assumed to be constant in the EMHD reconstruction, is 1.0 mV/m, which is comparable to 0.3 mV/m expected for the dimensionless reconnection rate 0.1 in the present case and those estimated for previously reported MP EDR events (Burch et al., 2020; Hasegawa et al., 2017). However, we show in Sections 3.3, 4.1, and 4.2 that the present MPCS probably involved 3D dynamics and time‐dependent or localized energy conversion processes; the measured electric field component $E_{zEMHD}$ was not stable at all.

**Figure 5:**
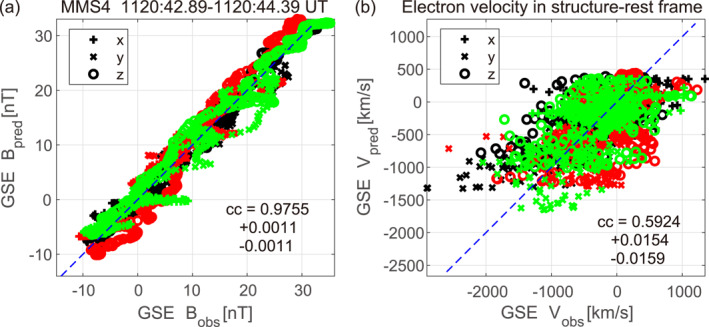
Correlations between the quantities predicted from the electron magnetohydrodynamics (EMHD) field maps (Figure 4) and those measured along the paths of the three spacecraft (MMS1, MMS2, and MMS3) not used in the reconstruction. GSM components of (a) the predicted versus measured magnetic fields, and (b) predicted versus measured electron velocities in the structure‐rest frame.

Figures 5a and 5b show scatter plots of the GSE components of the magnetic field and electron velocity in the structure frame, respectively, predicted from the MMS4 maps (Figure 4) versus those measured, along the MMS1, MMS2, and MMS3 paths. The correlation coefficient for the magnetic field ${cc}_{B}=0.9755$ is high, despite the fact that different spacecraft traversed different portions of the MPCS and the structure appears to have been evolving, as will be discussed in Section 3.3. This indicates that the reconstructed magnetic field (Figure 4a) represents some real features. On the other hand, the correlation coefficient for the electron velocity ${cc}_{V}=0.5924$ is much lower than ${cc}_{B}$. This is probably because errors in the reconstruction of electron streamlines are generally much larger than for the in‐plane magnetic field (Hasegawa et al., 2021) and the velocity structure was more 3D than the magnetic field. We note that ${cc}_{V}$ becomes even lower when the stream function ψ at y=0 is derived from integration along x of measured $n_{e}v_{y}$, rather than from measured $B_{z}$ (Hasegawa et al., 2017, 2019). This demonstrates that the lower correlation in Figure 5b is not due to the fact that the predicted in‐plane electron velocity components are computed from the reconstructed $B_{z}$ (and density). Interestingly, while MMS1 and MMS4 traversed nearly the same portion of the 2D field (Figure 4a) and observed very similar magnetic field patterns (Figures 3a–3c), consistent with 2D magnetic field, the electron velocities measured by MMS1 and MMS4 have different directions in substantial portions of the interval (Figures 3g–3i and 4b). Considering the fact that MMS1 and MMS4 were separated by 14.2 km ($∼6d_{e}$) in the out‐of‐plane ($z_{EMHD}$) direction (Table S1 in Supporting Information S1), our results suggest that the velocity field had significant 3D structures of electron scale.

### 3.3 Evolution of the MPCS

**Figure 6:**
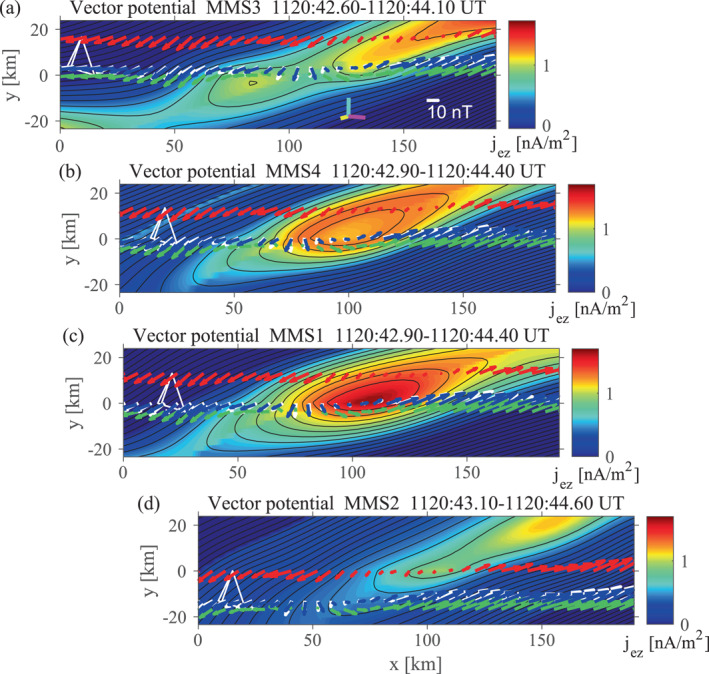
Magnetic field maps from the electron magnetohydrodynamics (EMHD) reconstruction applied individually to MMS3, MMS4, MMS1, and MMS2 data, respectively, shown in the order of current sheet crossing (Figure 3a). The format of each panel is the same as of Figure 4a. The panels for later crossings are shifted to the right, considering the different crossing times and reconstructed intervals (Figure 3a).

Figure 6 shows 2D magnetic field maps from all four spacecraft, plotted in the order of MPCS crossing (Figure 3a) and using the same frame velocity and coordinate system as for Figure 4. While the reconstructed field structure is similar for MMS4 and MMS1 which crossed the MPCS nearly at the same time, those for MMS2 and MMS3 are distinct from those for the other two. We argue that this difference is due to time evolution of the structure, rather than structures in the $z_{EMHD}$ direction. Note that MMS2 and MMS3 were located at a similar $z_{EMHD}$ position between MMS1 and MMS4 (Table S1 in Supporting Information S1). If the difference was due to 3D effects, MMS2 and MMS3 at similar $z_{EMHD}$ may have seen similar patterns in the field variations and MMS1 and MMS4 at largest (14.2 km) separation in $z_{EMHD}$ may have seen rather different patterns. On the contrary, MMS1 and MMS4 saw similar magnetic field variations and MM2 and MMS3 saw quite different variation patterns (Figures 3a–3c). It thus seems fair to conclude that there was a significant time evolution of the MPCS during a 0.5‐s period of the crossing (Figure 3a), which is much longer than electron gyroperiod ($1.4\times 10^{-3}$ s) and somewhat shorter than proton gyroperiod (2.5 s).

Figure 6 suggests that at the time when MMS3 crossed the MPCS, a small (sub‐ion‐scale) FR existed (Figure 6a), it grew to become an ISFR when MMS4 and MMS1 crossed the MPCS (Figures 6b and 6c), and it was swept toward the north (right side in the map) in the reconstruction frame by the time MMS2 crossed the MPCS (Figure 6d). Note, however, that the FR actually traveled southward in the spacecraft (or Earth's rest) frame, as is evident from the negative‐then‐positive $B_{N}$ (Figure 3c). MMS3 appears to have crossed a thinner part of the MPCS (Figure 6a) probably near the site of secondary reconnection, as will be discussed in Section 4.1. An enhanced electron velocity component $v_{eM}>2000$ km s^−1^, about one third of $V_{eA}$, was seen by MMS3 right at the center of the MPCS with $B_{L}∼0$ (at ∼43.5 s in Figure 3h). This indicates that a current sheet with half thickness ∼3 $d_{e}$ (∼7 km) existed at the MPCS center and near MMS3, which is also consistent with the field map from the MMS3 data (Figure 6a). Thus, the results suggest that the ISFR formed from an ECS in the MP, although MMS was not very near the primary X‐line (Section 4.1). On the other hand, such intense $v_{eM}$ seen by the other three spacecraft generally occurred off the MPCS center (Figure 3h), which suggests that those spacecraft saw ECSs at the boundary or separatrix regions of the developed ISFR. This interpretation is consistent with the reconstruction results shown in Figure 6.

**Figure 7:**
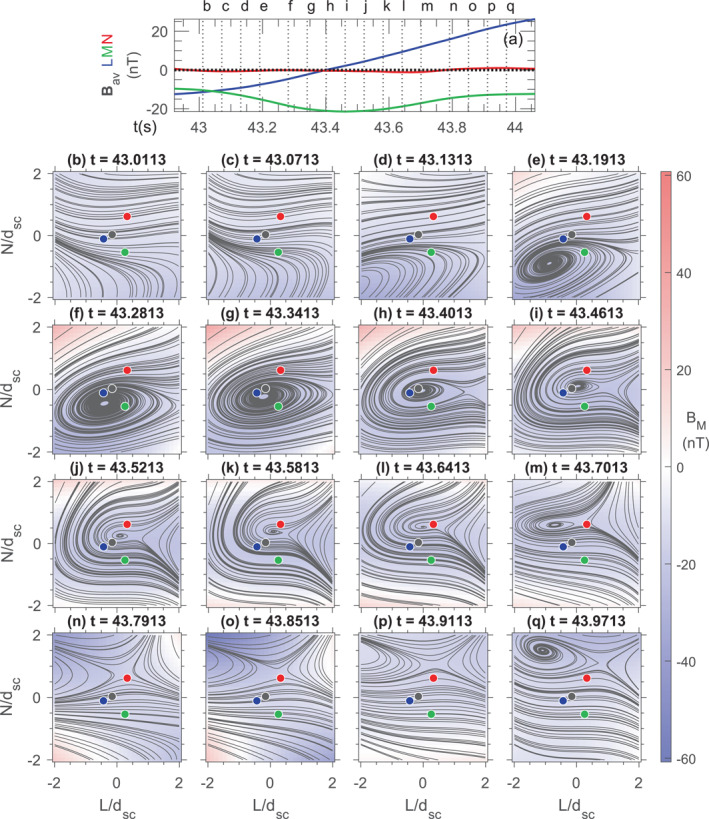
2D representation in the LN plane of 3D magnetic fields reconstructed for the interval 11:20:43–11:20:44 UT from the quadratic (Q‐3D) expansion of the polynomial reconstruction using multiple input times as input (Denton et al., 2022). (a) Four‐spacecraft average of the magnetic field in LMN (same as Figure S1a in Supporting Information S1). (b–q) Streamlines of the reconstructed magnetic field and $B_{M}$ in color for the moments marked in panel (a). The LMN coordinate system is the same as in Figure S1 in Supporting Information S1, and the angle between $M_{h}$ and the invariant axis ${\hat{z}}_{EMHD}$ is 20.9°.

Figure 7 shows 3D magnetic field lines in the L‐N plane reconstructed for each moment in time from the polynomial reconstruction (Denton et al., 2022), using the four‐spacecraft measurements of the magnetic field and current density from multiple times. We may rely on the reconstruction results within ∼2$d_{sc}$ of the MMS centroid, where $d_{sc}$ is the average spacecraft separation (∼15 km) (Denton et al., 2020). Here, instead of using the structure velocity that optimized the polynomial reconstruction, we used the velocity from the Shi et al. (2006, 2019) STD method determined in the following way. The full vector velocity was solved for except at times for which the minimum MDDB (Shi et al., 2005, 2019) eigenvalue was less that 50(0.1 nT/$d_{sc}$)^2^, where 0.1 nT is the maximum calibration error of the MMS magnetometer instruments (Russell et al., 2016). In that case the minimum gradient velocity component was set equal to zero. The resulting STD velocity was then averaged over 0.18 s, which was the time span used as input to the polynomial reconstruction.

A sequence of FR evolution appears different from that inferred from the EMHD reconstruction (Figure 6); Figure 7 suggests that the FR was shrinking in size for part of the interval. However, the polynomial reconstruction also suggests that an ion‐scale or sub‐ion‐scale FR existed in the MPCS when the MMS tetrahedron was near the center of the MPCS, and it moved southward (in the negative L direction) toward the end of the interval. In summary, both the EMHD and polynomial reconstructions suggest that the ISFR was generated from ECS reconnecting at the MP.

## 4 Discussion

### 4.1 Three‐Dimensional Effects

While the reconstruction and MDDB results suggest that the local magnetic structure in the MPCS was roughly 2D, the observed ISFR shows features inconsistent with expectations from 2D simulations (Nakamura et al., 2021) or 3D simulation with periodic boundary (Chen et al., 2012) of ISFRs, as will be discussed below. These simulations show that the electron density has a peak at the center of ISFRs, thus the electric field is intense around and points toward the ISFR center (Chen et al., 2012), and the electron temperature is strongly enhanced around the ISFR center, in particular in the parallel direction (Nakamura et al., 2021).

**Figure 8:**
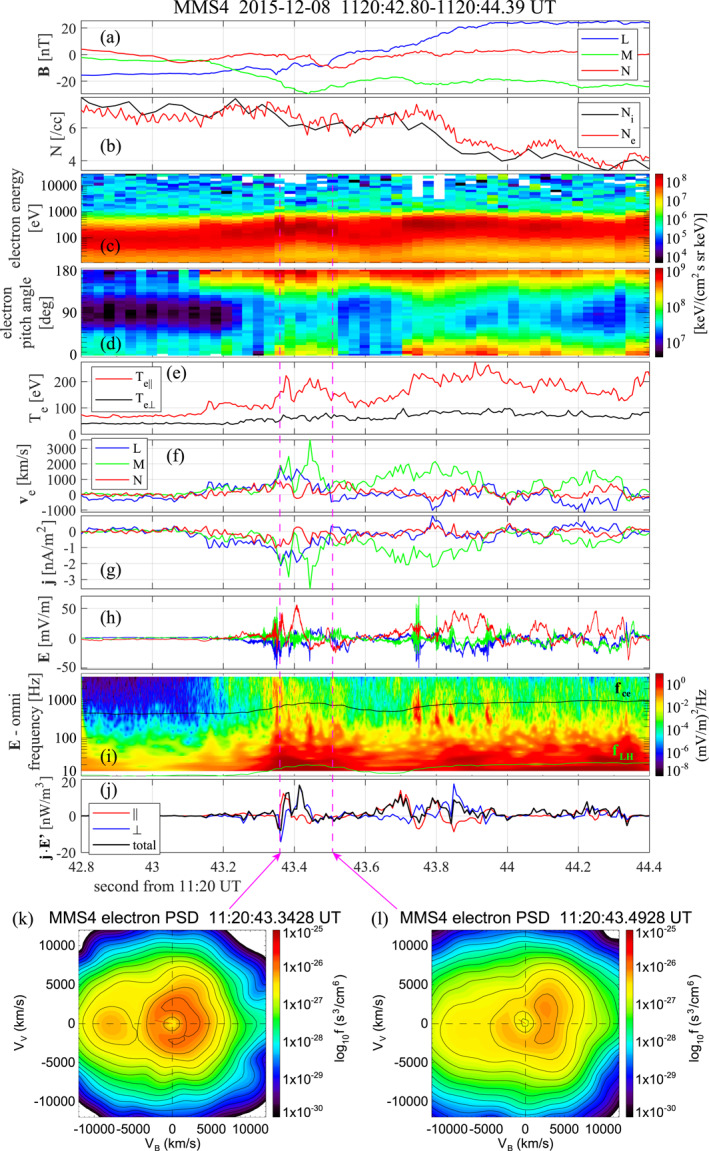
Burst‐mode MMS4 data around the reconstructed ion‐scale flux rope (Figure 4). (a) Magnetic field in LMN coordinates used in the reconstruction, (b) ion and electron densities with 37.5 and 7.5 ms resolutions, respectively, (c) energy versus time spectrogram of omnidirectional electrons, (d) pitch‐angle distributions of electrons with energies 0.2–2 keV, (e) electron temperatures parallel and perpendicular to the magnetic field, (f) electron velocity, (g) particle current density, (h) electric field data with 8.2 kHz resolution, (i) wavelet power spectrogram of the electric field, and (j) parallel and perpendicular component contributions to the electron‐frame energy conversion rate $j\cdot E^{\prime }$ and total magnitude with 7.5 ms resolution. The black and green curves in panel (i) mark the electron cyclotron and lower‐hybrid frequencies ($f_{ce}$ and $f_{LH}$, respectively). (k and l) Electron velocity distributions in the BV plane with field‐aligned velocity as the horizontal axis and perpendicular velocity as the vertical axis, at times marked by the magenta dashed lines. Electron energy‐time spectrogram (c), pitch‐angle distribution (d), electric field power spectrum (i), and electron velocity distributions (k and l) were created by SPEDAS (Angelopoulos et al., 2019), whereas the other panels by Matlab.

Figure 4e shows the measured parallel electron temperatures plotted along the paths of the four spacecraft (see Figure S2 in Supporting Information S1 to clearly see the temperature seen by MMS1), and Figure 8 shows data from various instruments, including the electric field probes, taken by MMS4 around the ISFR interval (the interval 11:20:42.8–11:20:44.4 UT shown in Figure 8 is nearly the same as that used in the EMHD reconstruction, as shown in Figures 4 and 6b). Whereas the parallel temperature is enhanced around the ISFR boundary, it has a local minimum around the ISFR center (Figure 4e and ∼43.6 s in Figure 8e). Moreover, the electron density shows no clear peak (Figure 8b), and the electric field is not intense (Figures 8h and 8i) around the ISFR center. Figure 8d shows that electrons at 0.2–2 keV energies (heated or accelerated electrons of magnetosheath origin) around the ISFR boundaries (∼43.4 and ∼43.8 s) were bi‐directional with more intense fluxes at pitch angles 0° and 180°. This is consistent with field‐line connection to two X‐lines and thus magnetic FR interpretation. On the other hand, 0.2–2 keV electrons had intense fluxes only at 180° around the ISFR center, suggesting connection to only one X‐line. This is contrary to expectation from a 2D FR picture. Similar electron pitch‐angle distribution signatures have been observed in MP FRs (Hasegawa et al., 2015; Øieroset et al., 2011; Pu et al., 2013; Zhong et al., 2013), indicating complex magnetic topology of FTEs. These features indicate that the observed ISFR had 3D effects and was not as simple as seen in 2D simulations (Daughton et al., 2006; Drake et al., 2006; Hesse et al., 1999; Nakamura et al., 2021).

**Figure 9:**
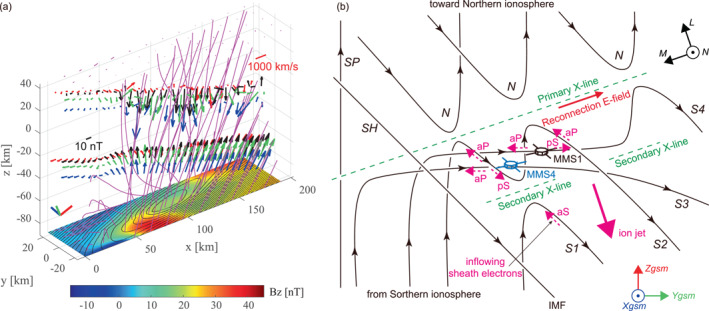
(a) Three‐dimensional view in reconstruction coordinates of the 2D magnetic field lines recovered from the electron magnetohydrodynamics (EMHD) reconstruction applied to MMS4 data (Figure 4). The black, red, green, and blue arrows in the negative *z* region are the vectors of magnetic fields measured by MMS1, MMS2, MMS3, and MMS4, respectively, and those in the positive *z* region are the vectors of the measured electron velocities in the structure frame. The blue, green, and red bars at lower left are the unit vectors of the GSM *x*, *y*, and *z* axes, respectively. (b) Schematic of possible magnetic topology and geometry of the ion‐scale flux rope (ISFR) on the subsolar magnetopause, as viewed from the sun, when MMS4 and MMS1 were near the center of the ISFR. “SH” and “SP” mark unreconnected magnetosheath and magnetospheric field lines, respectively. “N” marks standard reconnected field lines on the northern side of the primary X‐line with connection to the northern ionosphere. “S1” and “S2” mark the field lines formed through reconnection at secondary X‐line between two standard reconnected field lines on the southern side of the primary X‐line. While “S1” was on the southern side of secondary X‐line, “S2” passed through the region inside, but near the boundary of the ISFR. “S3” is the field line that experienced reconnection at the primary X‐line and passed through the central region of the ISFR, and “S4” is the field line that experienced reconnection at both the primary and secondary X‐lines and passed through the ISFR central region. Magenta dashed arrows indicate magnetosheath electron populations inflowing toward either primary or secondary X‐lines, with “aP” and “aS” representing electrons streaming antiparallel to the magnetic field toward the primary and secondary X‐lines, respectively, and “pS” the electrons streaming parallel to the field toward secondary X‐line.

The magnetosheath‐side separatrix appears to have been encountered by MMS4 at ∼43.15 s, after which the parallel temperature increased (Figure 8e), antiparallel streaming electrons were observed throughout, and positive $v_{eL}$ was observed (Figures 4b and 8f) (until ∼43.5 s, which was slightly before the MPCS center was crossed). Note that in Figure 4b the direction of spacecraft motion and L are in the positive *x* direction. We interpret this positive $v_{eL}$ as electron inflow on the magnetosheath side toward the primary X‐line that would have been continuously active and longer than ion scales on the northern side of MMS. This interpretation is consistent with exclusively southward ion jets not only during the FTE intervals but even after the MPCS crossing and also with duskward ion flow on the magnetospheric side (Figure 1b), which is expected on the southern side of X‐line under duskward magnetosheath field conditions (Figure 9b).

Figures 8k and 8l show examples of electron velocity distributions with bidirectional streaming signatures observed on the magnetosheath side. We interpret anti‐field‐aligned streaming electrons, observed continuously on the magnetosheath side of the MPCS center but on the earthward side of the magnetosheath separatrix (Figure 8d), as those inflowing toward the primary X‐line on the northern side of MMS (“aP” on field line “S2” in Figure 9b). Field‐aligned electrons, observed only transiently on the magnetosheath side, would be those streaming toward southern, secondary X‐line (“pS” on field line “S2”). Asymmetries in speed and phase space density between the field‐aligned and anti‐field‐aligned electrons are likely to be guide‐field effects; in the present event, the guide field intensity ∼20 nT was comparable to that of the reconnecting field (Figure 3). In the presence of guide field with negative $B_{M}$ (Figures 8a and 9b), electrons streaming antiparallel to the magnetic field (i.e., roughly in the direction antiparallel to the reconnection electric field which is in the −M direction) are more strongly sucked into the primary reconnection region, producing a density minimum, and are more strongly accelerated than parallel streaming electrons (Pritchett & Coroniti, 2004). Hall magnetic field signature (negative increase of $B_{M}$ in Figure 7a) and density dip, which is not very clear in Figure 7b but is obvious for MMS2 and MMS3 (Figures S4b and S5b in Supporting Information S1), support our interpretation. Note that a density dip is a signature of magnetosheath‐side separatrix regions for asymmetric, guide‐field reconnection (Figure 1 in Choi et al., 2022).

We also note that temporal variations around the ISFR center of the electron temperature and pitch‐angle distribution seen by MMS1, which was 14.2 km ($∼6d_{e}$) away from MMS4 in $z_{EMHD}$ (Table S1 in Supporting Information S1), differed significantly from those seen by MMS4. Comparison between Figure 8 and Figure S3 in Supporting Information S1 suggests that around the ISFR center, MMS4 more often encountered a type “S3” field line, with only anti‐field‐aligned magnetosheath electrons, while MMS1 more often encountered a type “S4” field line with bidirectional electrons (see Figure 9b for types of field line). This is consistent with electron‐scale 3D structures in the ISFR axial or X‐line direction.

These features suggest that the primary X‐line on the northern side of MMS was persistently active, but secondary X‐line that contributed to the formation of the 3D ISFR in the MPCS was patchy or transiently active (Figure 9b). The secondary X‐line(s) could have been of sub‐ion scale, because no northward ion jet or acceleration was observed (Figure 1b). Figure 9a shows 3D representation of the 2D magnetic field lines from the EMHD reconstruction for MMS4 (Figure 4), along with the magnetic field and electron velocity vectors measured by the four spacecraft. One can expect that under significant guide field conditions, as in the present case, field lines may be connected to regions distant in the FR axial direction where reconnection may or may not be active. Figure 9a also shows that while electron flows in and around the ISFR were roughly antiparallel to the magnetic field, they were less organized and more different among the four spacecraft than the magnetic field. Such a feature is consistent with 3D and complex topology or connectivity of the ISFR field lines, as depicted in Figure 9b. The suggested patchy or transient X‐line(s) on the sub‐ion scale is consistent with 3D fully kinetic simulations of magnetic reconnection with guide field, as reported by Daughton et al. (2011) and Nakamura et al. (2016), in which turbulent reconnection generates 3D filamentary ion‐scale flux ropes. On the other hand, turbulent fluctuations of the upstream magnetosheath magnetic field may also have led to the localized and/or intermittent nature of secondary X‐line(s) (Genestreti et al., 2022).

### 4.2 Origin of Positive and Negative $j\cdot \left(E+v_{e}\times B\right)$

Figure 4d shows the electron‐frame energy conversion rate $j\cdot E^{\prime }=j\cdot \left(E+v_{e}\times B\right)$ plotted along the paths of the four spacecraft. As reported by Burch et al. (2018), both positive and negative $j\cdot E^{\prime }$ were observed, indicating that electromagnetic energy was being converted to electron energy in some parts of the MPCS and vice versa in other parts. An interesting feature is that many energy conversion regions were distributed in separatrix regions of the ISFR and on both magnetosheath and magnetospheric sides. Note that the magnitude of the energy conversion rates is much larger ($\geq 10\;nW/m^{3}$) than predicted ($\leq 4\;nW/m^{3}$) in the EDRs of MP reconnection, as pointed out by Burch et al. (2018), and those energy conversion regions appear far from X‐line(s) (Figure 4d and Figure S2d in Supporting Information S1).

Figures 8d, 8e, 8h, and 8j (see also Figures S3–S5 in Supporting Information S1 for the spacecraft other than MMS4) show that many of the large amplitude $j\cdot E^{\prime }$ regions are collocated with large amplitude electric fields and high parallel electron temperature associated with bi‐directional field‐aligned electrons. The large amplitude electric fields were mostly of electrostatic fluctuations with frequencies below, around, or above the electron cyclotron frequency $f_{ce}$, many with broadband features (Figure 8i). Burch et al. (2018) reported that some fluctuations observed around the magnetosheath separatrix were of oblique weakly electromagnetic whistler waves with frequencies around $f_{ce}$ (their Fig. 3). While the generation mechanisms of these intermediate‐frequency waves (Khotyaintsev et al., 2019) are beyond the scope of the present paper and need further investigation, it is probable that they were driven by bi‐directional electron beams (Wang et al., 2022), as shown in Figures 8k and 8l. A number of simulations and observations have shown that electron beam‐driven or two‐stream instabilities and resulting electrostatic waves (EWs), electrostatic solitary waves (ESWs or electron holes), or whistler waves can be responsible for electrostatic and electromagnetic fluctuations around $f_{ce}$ in separatrix regions (Choi et al., 2022; Fujimoto, 2014; Goldman et al., 2014; Holmes et al., 2019; Steinvall et al., 2019).

Figure 8j (see also Figures S3–S5 in Supporting Information S1) shows that the parallel component contribution to $j\cdot E^{\prime }$, $j_{∥}E_{∥}$, was positive in some regions and negative in other regions. Some intense positive $j_{∥}E_{∥}$ (e.g., at ∼43.37 s) was observed without much electric field fluctuations, and thus may be due to field‐aligned (electrostatic potential) acceleration of electrons inflowing toward the primary X‐line (Egedal et al., 2015). Other positive $j_{∥}E_{∥}$ (e.g., at ∼43.8 and ∼43.95 s) coexist with intermediate‐frequency electrostatic fluctuations, and may be associated with electrostatic wave to electron energy conversion, namely, electron heating. Interestingly, some intense negative $j_{∥}E_{∥}$ (at ∼43.75 and ∼43.83 s in Figure 8j) coexisted with intermediate‐frequency electrostatic fluctuations, and may be an indication of electron beam energy being converted to energy of EWs or ESWs.

An intense positive $j_{∥}E_{∥}$ was seen by MMS4 at ∼43.7 s, when no prominent intermediate‐frequency electric field fluctuations were observed (Figures 8h–8j). This is possibly associated with energy conversion of actual MP reconnection in the presence of guide field. However, its magnitude (∼10 $nW/m^{3}$) was much larger than expected for standard MP reconnection (Burch et al., 2018), and it was seen near the ISFR center (Figure 4d) that may have been connected to a portion of the primary X‐line (Figure 9). We also note that such intense $j\cdot E^{\prime }$ was not seen by MMS1 near the ISFR center (Figure S3d in Supporting Information S1). Thus, it might be a signature of patchy or bursty reconnection (Genestreti et al., 2022). Moreover, intense positive and negative $j_{⊥}\cdot E_{⊥}^{\prime }$ were seen at ∼43.4 and ∼43.84 s with no significant intermediate frequency waves. Since they were observed in density gradient regions, it is possible that they were associated with lower‐hybrid waves excited around the separatrices (Marshall et al., 2022; Pritchett, 2013). Further investigation of these features is necessary.

A unique feature of the present event is that both bi‐directional electron beams and large‐amplitude electric field fluctuations were observed not only in the magnetospheric but also in the magnetosheath separatrix regions, while EWs and ESWs themselves have been observed on the magnetosheath side of MP reconnection layers (Graham et al., 2015, 2016; Zhong, Graham et al., 2021). This would be because of the presence of ISFR with connection to two X‐lines. Note that those in magnetospheric separatrix regions are common (Hwang et al., 2017; Khotyaintsev et al., 2020; Wilder et al., 2019, 2017), and this is attributed partially to higher speeds on the magnetospheric side of magnetosheath‐origin electrons as they are accelerated in the EDR by the reconnection electric field before entering into magnetospheric separatrix regions (in addition to acceleration in magnetosheath separatrix regions) (e.g., Choi et al., 2022).

In summary, the origin of oscillatory or positive and negative localized $j\cdot E^{\prime }$ appears to be in the separatrix regions, associated with intermediate‐frequency waves (EWs, ESWs, or double layers) generated by bi‐directional electron beams or lower‐hybrid waves excited in density gradient regions (Graham et al., 2019; Marshall et al., 2022). It is also possible that 3D or transient nature of MP reconnection in the presence of guide field (Daughton et al., 2011) or magnetosheath turbulence (Genestreti et al., 2022), as discussed in Section 4.1, was responsible for them. The results suggest that while intense $j\cdot E^{\prime }$ can be used as an indicator of active reconnection not far from the observation site, it may not necessarily indicate electron‐scale proximity to EDRs of major magnetic topology change. Note, however, that the field‐line connectivity can change in double layer‐like structures (Schindler et al., 1988), which can exist in separatrix regions and may exhibit intense $j_{∥}E_{∥}>0$ features (Ergun, Goodrich, et al., 2016; Ergun, Tucker, et al., 2016).

### 4.3 Generation and Decay Processes of FTEs

We argue that the three FTEs preceding the MPCS were produced by the same mechanism that generated the ISFR in the MPCS, namely, secondary reconnection. Table 1 shows the HT velocity, which can be taken as the FTE structure velocity, the axial orientations of the three FTEs, and their angles relative to the ISFR axis. It is seen that the FR2 and FR3 axes were relatively close to the ISFR axis, and the southward component of the HT velocity was larger for FR3, closest to the reconnecting MPCS, than for FR1. These results are consistent with FTE generation near the primary X‐line that formed the ISFR, and suggest that the FTEs initially had higher speeds near the X‐line but were decelerated as they traveled southward and plowed through ambient plasmas. Thus, our analysis supports the conclusion by Dong et al. (2017) that FTEs can form through secondary magnetic reconnection in elongated ECSs (Daughton et al., 2006; Drake et al., 2006). The FR1 axial direction is rather different. This is likely because FR1 was observed not near the current sheet center but in the magnetosheath (Figure 1a). Note that the single‐spacecraft method for FR axis estimation (Hu & Sonnerup, 2002) tends to give axial orientations closer to the average magnetic field direction for the reconstruction interval, that is the period when an FTE is encountered.

We also point out that another, small‐scale FTE was observed by MMS at 11:20:40 UT between FR3 and the MP crossing (Figure 1). Although short in duration, it had typical signatures of southward‐moving FTEs, such as negative‐then‐positive $B_{x}$ and enhancements in the field intensity and total pressure (Figures 1a and 1g). We could not reconstruct this FTE because it was encountered in the magnetosheath region with negatively large $B_{z}$ away from the current sheet. However, it involved fast northward electron flows (Figure 1c), probably associated with electron inflow toward the primary X‐line. It is thus likely that different stages of FTE evolution were observed in the present single MMS event, from sub‐ion‐scale or ion‐scale FR to smaller and then larger FTEs.

Our study suggests that ion‐scale flux ropes and meso‐scale FTEs (on ion‐to‐MHD scales smaller than typical FTEs with sizes ∼1$R_{E}$) (Hasegawa et al., 2016) can be generated from ECSs with single primary X‐line but with secondary X‐line(s). However, we do not claim that all FTEs can be generated by the same mechanism, that is, from single primary X‐line. There are at least two types of MP FTEs: one type embedded in unidirectional reconnection ion jets, and the other sandwiched between oppositely directed, colliding reconnection ion jets. The former type is those seen in Figure 1 and as reported by Phan et al. (2004) and Hasegawa et al. (2016) that appear to be observed during continuous MP reconnection. The latter type was reported by Hasegawa et al. (2010) and Øieroset et al. (2011) and may require more than one X‐lines with MHD‐scale separation from each other (otherwise no colliding ion jets) or sequential formation of primary X‐lines (Raeder, 2006). There may also be FTEs formed through intermittent bursts of MP reconnection at single X‐line (Fear et al., 2012).

Hasegawa et al. (2016) suggested, based on simultaneous Geotail and MMS observations of reconnection jets at the dayside MP at low‐ and mid‐latitudes, respectively, that meso‐scale FTEs may decay during the course of poleward propagation. As reported recently by Øieroset et al. (2019, 2016), Kacem et al. (2018), Fargette et al. (2020), Qi et al. (2020), and Zhong, Zhou et al. (2021), magnetic flux tubes and energy in meso‐scale or ion‐scale FRs may be disentangled and dissipated, respectively, by magnetic reconnection inside or at the boundary of FTEs. These FTE decay processes may contribute to the formation of the low‐latitude boundary layer (Nishida, 1989) and a collection of such processes may affect dayside to nightside flux transfer on the large scale. Thus, further exploration is needed of FTE evolution processes.

## 5 Summary

We have analyzed three FTEs and a subsequent sub‐ion‐scale MPCS, which was reconnecting in the presence of significant guide field and showed intense positive and negative values of $j\cdot E^{\prime }$, observed by the MMS spacecraft on 8 December 2015. The results can be summarized as follows:

1. The reconstructions of the MPCS by both the EMHD and polynomial reconstruction methods suggest that an ISFR existed in the MPCS and was likely growing from an ECS, consistent with ISFR generation by secondary magnetic reconnection.
2. GSR shows that the axial orientations of two of the three FTE FRs that were closer to the MPCS than the other were similar to that of the ISFR, suggesting the same generation mechanism, that is, secondary reconnection in ECS.
3. While bi‐directional electron beams observed in separatrix regions of the ISFR are consistent with X‐lines on both the northern and southern sides of the ISFR, unidirectional electrons consistent with connection only to the northern X‐line were observed around the ISFR center. This suggests three‐dimensional magnetic topology of the observed ISFR. The localized or transient nature of intense $j\cdot E^{\prime }>0$ around the ISFR center, seen by MMS4 but not by other spacecraft, is consistent with such interpretation. It is likely that secondary reconnection in the present event with significant guide field was patchy or intermittent.
4. Most of intense oscillatory energy conversion features were observed in separatrix regions of the ISFR, many collocated with bi‐directional electron beams and/or large‐amplitude electric field fluctuations suggestive of electrostatic waves or structures (solitary waves or double layers). In the present event, intense $j\cdot E^{\prime }$ appears to be mostly due to activities in the separatrix regions, rather than to energy conversion in the EDR.
5. The last point implies that while significant $j\cdot E^{\prime }$ may be an indication of active reconnection site not far from the observation site, it may not necessarily indicate close (electron‐scale) vicinity to region(s) of major magnetic topology change. Spacecraft measurements with multi‐scale separations would be needed to reveal time history of multi‐dimensional and cross‐scale energy conversion and transfer phenomena and effects of the upstream and asymmetric boundary conditions (Genestreti et al., 2022).

A final remark is that while the present EMHD reconstruction assumes isotropy of the gyrotropic part of electron velocity distributions (Hasegawa et al., 2021), strong electron temperature anisotropy is common in reconnection layers (Figures 1e and 8e), in particular, in the presence of guide field (e.g., Wetherton et al., 2022). Further improvement of the EMHD method is thus necessary to incorporate electron pressure anisotropy effects.

## Supporting information

Supporting Information S1

## Data Availability

All MMS data used in this study are publicly available via the MMS Science Data Center at https://lasp.colorado.edu/mms/sdc/public/about/browse-wrapper/. All code used to produce the MMS data analyzed in this study is based on the publicly available SPEDAS tools (Angelopoulos et al., 2019) (http://spedas.org/downloads/spedas_5_0.zip). The Matlab code for the EMHD reconstruction can be found at the Zenodo (https://doi.org/10.5281/zenodo.5144478), while that for the Grad‐Shafranov reconstruction is available at GitHub (https://github.com/cmoestl/interplanetary-grad-shafranov). The Matlab code for polynomial reconstruction using data from multiple times, used for producing Figure 7, is available in a Zenodo repository (https://doi.org/10.5281/zenodo.6941597).

## References

[jgra57717-bib-0001] Angelopoulos, V. , Cruce, P. , Drozdov, A. , Grimes, E. W. , Hatzigeorgiu, N. , King, D. A. , et al. (2019). The space physics Environment data analysis system (SPEDAS). Space Science Reviews, 215(1), 9. 10.1007/s11214-018-0576-4 30880847 PMC6380193

[jgra57717-bib-0002] Burch, J. L. , Ergun, R. E. , Cassak, P. A. , Webster, J. M. , Torbert, R. B. , Giles, B. L. , et al. (2018). Localized oscillatory energy conversion in magnetopause reconnection. Geophysical Research Letters, 45(3), 1237–1245. 10.1002/2017GL076809

[jgra57717-bib-0003] Burch, J. L. , & Phan, T. D. (2016). Magnetic reconnection at the dayside magnetopause: Advances with MMS. Geophysical Research Letters, 43(16), 8327–8338. 10.1002/2016GL069787

[jgra57717-bib-0004] Burch, J. L. , Torbert, R. B. , Phan, T. D. , Chen, L. J. , Moore, T. E. , Ergun, R. E. , et al. (2016). Electron‐scale measurements of magnetic reconnection in space. Science, 352(6290), aaf2939. 10.1126/science.aaf2939 27174677

[jgra57717-bib-0005] Burch, J. L. , Webster, J. M. , Hesse, M. , Genestreti, K. J. , Denton, R. E. , Phan, T. D. , et al. (2020). Electron inflow velocities and reconnection rates at Earth’s magnetopause and magnetosheath. Geophysical Research Letters, 47(17), e2020GL089082. 10.1029/2020GL089082

[jgra57717-bib-0006] Chen, L.‐J. , Daughton, W. , Bhattacharjee, A. , Torbert, R. B. , Roytershteyn, V. , & Bessho, N. (2012). In‐plane electric fields in magnetic islands during collisionless magnetic reconnection. Physics of Plasmas, 19(11), 112902. 10.1063/1.4767645

[jgra57717-bib-0007] Choi, S. , Bessho, N. , Wang, S. , Chen, L.‐J. , & Hesse, M. (2022). Whistler waves generated by nongyrotropic and gyrotropic electron beams during asymmetric guide field reconnection. Physics of Plasmas, 29(1), 012903. 10.1063/5.0059884

[jgra57717-bib-0008] Daughton, W. , Roytershteyn, V. , Karimabadi, H. , Yin, L. , Albright, B. J. , Bergen, B. , & Bowers, K. J. (2011). Role of electron physics in the development of turbulent magnetic reconnection in collisionless plasmas. Nature Physics, 7, 539–542. 10.1038/nphys1965

[jgra57717-bib-0009] Daughton, W. , Scudder, J. , & Karimabadi, H. (2006). Fully kinetic simulations of undriven magnetic reconnection with open boundary conditions. Physics of Plasmas, 13(7), 072101. 10.1063/1.2218817

[jgra57717-bib-0010] Denton, R. E. , Liu, Y.‐H. , Hasegawa, H. , Torbert, R. B. , Li, W. , Fuselier, S. , & Burch, J. L. (2022). Polynomial reconstruction of the magnetic field observed by multiple spacecraft with integrated velocity determination. Journal of Geophysical Research: Space Physics, 127(10), e2022JA030512. 10.1029/2022JA030512

[jgra57717-bib-0011] Denton, R. E. , Sonnerup, B. U. Ö. , Russell, C. T. , Hasegawa, H. , Phan, T.‐D. , Strangeway, R. J. , et al. (2018). Determining L‐M‐N current sheet coordinates at the magnetopause from Magnetospheric Multiscale data. Journal of Geophysical Research: Space Physics, 123(3), 2274–2295. 10.1002/2017JA024619

[jgra57717-bib-0012] Denton, R. E. , Torbert, R. B. , Hasegawa, H. , Dors, I. , Genestreti, K. J. , Argall, M. R. , et al. (2020). Polynomial reconstruction of the reconnection magnetic field observed by multiple spacecraft. Journal of Geophysical Research: Space Physics, 125(2), e2019JA027481. 10.1029/2019JA027481

[jgra57717-bib-0013] Dong, X.‐C. , Dunlop, M. W. , Trattner, K. J. , Phan, T. D. , Fu, H. , Cao, J. , et al. (2017). Structure and evolution of flux transfer events near dayside magnetic reconnection dissipation region: MMS observations. Geophysical Research Letters, 44(12), 5951–5959. 10.1002/2017GL073411

[jgra57717-bib-0014] Drake, J. F. , Swisdak, M. , Schoeffler, K. M. , Rogers, B. N. , & Kobayashi, S. (2006). Formation of secondary islands during magnetic reconnection. Geophysical Research Letters, 33(13), L13105. 10.1029/2006GL025957

[jgra57717-bib-0015] Eastwood, J. P. , Phan, T. D. , Cassak, P. A. , Gershman, D. J. , Haggerty, C. , Malakit, K. , et al. (2016). Ion‐scale secondary flux ropes generated by magnetopause reconnection as resolved by MMS. Geophysical Research Letters, 43(10), 4716–4724. 10.1002/2016GL068747 27635105 PMC5001194

[jgra57717-bib-0016] Egedal, J. , Daughton, W. , Le, A. , & Borg, A. L. (2015). Double layer electric fields aiding the production of energetic flat‐top distributions and superthermal electrons within magnetic reconnection exhausts. Physics of Plasmas, 22(10), 101208. 10.1063/1.4933055

[jgra57717-bib-0017] Ergun, R. E. , Goodrich, K. A. , Wilder, F. D. , Holmes, J. , Stawarz, J. , Eriksson, S. , et al. (2016). Magnetospheric Multiscale satellites observations of parallel electric fields associated with magnetic reconnection. Physical Review Letters, 116(23), 235102. 10.1103/PhysRevLett.116.235102 27341241

[jgra57717-bib-0018] Ergun, R. E. , Tucker, S. , Westfall, J. , Goodrich, K. A. , Malaspina, D. M. , Summers, D. , et al. (2016). The axial double probe and fields signal processing for the MMS mission. Space Science Reviews, 199(1–4), 167–188. 10.1007/s11214-014-0115-x

[jgra57717-bib-0019] Fargette, N. , Lavraud, B. , Øieroset, M. , Phan, T. D. , Toledo‐Redondo, S. , Kieokaew, R. , et al. (2020). On the ubiquity of magnetic reconnection inside flux transfer event‐like structures at the Earth’s magnetopause. Geophysical Research Letters, 47(6), e2019GL086726. 10.1029/2019GL086726

[jgra57717-bib-0020] Fear, R. C. , Milan, S. E. , & Oksavik, K. (2012). Determining the axial direction of high‐shear flux transfer events: Implications for models of FTE structure. Journal of Geophysical Research, 117(A9), A09220. 10.1029/2012JA017831

[jgra57717-bib-0021] Fujimoto, K. (2014). Wave activities in separatrix regions of magnetic reconnection. Geophysical Research Letters, 41(8), 2721–2728. 10.1002/2014GL059893

[jgra57717-bib-0022] Genestreti, K. J. , Burch, J. L. , Cassak, P. A. , Torbert, R. B. , Ergun, R. E. , Varsani, A. , et al. (2017). The effect of a guide field on local energy conversion during asymmetric magnetic reconnection: MMS observations. Journal of Geophysical Research: Space Physics, 122(11), 11342–11353. 10.1002/2017JA024247

[jgra57717-bib-0023] Genestreti, K. J. , Li, X. , Liu, Y.‐H. , Burch, J. L. , Torbert, R. B. , Fuselier, S. A. , et al. (2022). On the origin of “patchy” energy conversion in electron diffusion regions. Physics of Plasmas, 29(8), 082107. 10.1063/5.0090275

[jgra57717-bib-0024] Goldman, M. V. , Newman, D. L. , Lapenta, G. , Andersson, L. , Gosling, J. , Eriksson, S. , et al. (2014). Cerenkov emission of quasiparallel whistlers by fast electron phase‐space holes during magnetic reconnection. Physical Review Letters, 112(14), 145002. 10.1103/PhysRevLett.112.145002 24765977

[jgra57717-bib-0025] Graham, D. B. , Khotyaintsev, Y. V. , Norgren, C. , Vaivads, A. , Andre, M. , Drake, J. F. , et al. (2019). Universality of lower hybrid waves at Earth’s magnetopause. Journal of Geophysical Research: Space Physics, 124(11), 8727–8760. 10.1029/2019JA027155

[jgra57717-bib-0026] Graham, D. B. , Khotyaintsev, Y. V. , Vaivads, A. , & Andre, M. (2015). Electrostatic solitary waves with distinct speeds associated with asymmetric reconnection. Geophysical Research Letters, 42(2), 215–224. 10.1002/2014GL062538

[jgra57717-bib-0027] Graham, D. B. , Khotyaintsev, Y. V. , Vaivads, A. , & Andre, M. (2016). Electrostatic solitary waves and electrostatic waves at the magnetopause. Journal of Geophysical Research: Space Physics, 121(4), 3069–3092. 10.1002/2015JA021527

[jgra57717-bib-0028] Hasegawa, H. (2012). Structure and dynamics of the magnetopause and its boundary layers. Monographs on Environment, Earth and Planets, 1(2), 71–119. 10.5047/meep.2012.00102.0071

[jgra57717-bib-0084] Hasegawa, H. , Denton, R. E. , Nakamura, R. , Genestreti, K. J. , Nakamura, T. K. M. , Hwang, K.‐J. , et al. (2019). Reconstruction of the electron diffusion region of magnetotail reconnection seen by the MMS spacecraft on 11 July 2017. Journal of Geophysical Research: Space Physics, 124, 122–138. 10.1029/2018JA026051

[jgra57717-bib-0029] Hasegawa, H. , Denton, R. E. , Nakamura, T. K. M. , Genestreti, K. J. , Phan, T. D. , Nakamura, R. , et al. (2022). Magnetic field annihilation in a magnetotail electron diffusion region with electron‐scale magnetic island. Journal of Geophysical Research: Space Physics, 127(7), e2022JA030408. 10.1029/2022JA030408 PMC9541864 36248013

[jgra57717-bib-0030] Hasegawa, H. , Kitamura, N. , Saito, Y. , Nagai, T. , Shinohara, I. , Yokota, S. , et al. (2016). Decay of mesoscale flux transfer events during quasi‐continuous spatially extended reconnection at the magnetopause. Geophysical Research Letters, 43(10), 4755–4762. 10.1002/2016GL069225

[jgra57717-bib-0031] Hasegawa, H. , Nakamura, T. K. M. , & Denton, R. E. (2021). Reconstruction of the electron diffusion region with inertia and compressibility effects. Journal of Geophysical Research: Space Physics, 126(11), e2021JA029841. 10.1029/2021JA029841 PMC9286637 35864949

[jgra57717-bib-0032] Hasegawa, H. , Sonnerup, B. U. Ö. , Denton, R. E. , Phan, T. , Nakamura, T. K. M. , Giles, B. L. , et al. (2017). Reconstruction of the electron diffusion region observed by the Magnetospheric Multiscale spacecraft: First results. Geophysical Research Letters, 44(10), 4566–4574. 10.1002/2017GL073163

[jgra57717-bib-0033] Hasegawa, H. , Sonnerup, B. U. Ö. , Eriksson, S. , Nakamura, T. K. M. , & Kawano, H. (2015). Dual‐spacecraft reconstruction of a three‐dimensional magnetic flux rope at the Earth’s magnetopause. Annales Geophysicae, 33(2), 169–184. 10.5194/angeo-33-169-2015

[jgra57717-bib-0034] Hasegawa, H. , Sonnerup, B. U. Ö. , Owen, C. J. , Klecker, B. , Paschmann, G. , Balogh, A. , & Rème, H. (2006). The structure of flux transfer events recovered from Cluster data. Annales Geophysicae, 24(2), 603–618. 10.5194/angeo-24-603-2006

[jgra57717-bib-0035] Hasegawa, H. , Wang, J. , Dunlop, M. W. , Pu, Z. Y. , Zhang, Q.‐H. , Lavraud, B. , et al. (2010). Evidence for a flux transfer event generated by multiple X‐line reconnection at the magnetopause. Geophysical Research Letters, 37(16), L16101. 10.1029/2010GL044219

[jgra57717-bib-0036] Hau, L.‐N. , & Sonnerup, B. U. Ö. (1999). Two‐dimensional coherent structures in the magnetopause: Recovery of static equilibria from single‐spacecraft data. Journal of Geophysical Research, 104(A4), 6899–6917. 10.1029/1999JA900002

[jgra57717-bib-0037] Hesse, M. , Schindler, K. , Birn, J. , & Kuznetsova, M. (1999). The diffusion region in collisionless magnetic reconnection. Physics of Plasmas, 6(5), 1781–1795. 10.1063/1.873436

[jgra57717-bib-0038] Holmes, J. C. , Ergun, R. E. , Nakamura, R. , Roberts, O. , Wilder, F. D. , & Newman, D. L. (2019). Structure of electron‐scale plasma mixing along the dayside reconnection separatrix. Journal of Geophysical Research: Space Physics, 124(11), 8788–8803. 10.1029/2019JA026974

[jgra57717-bib-0039] Hu, Q. , & Sonnerup, B. U. Ö. (2002). Reconstruction of magnetic clouds in the solar wind: Orientations and configurations. Journal of Geophysical Research, 107(A7), 1142. 10.1029/2001JA000293

[jgra57717-bib-0040] Hu, Q. , & Sonnerup, B. U. Ö. (2003). Reconstruction of two‐dimensional structures in the magnetopause: Method improvements. Journal of Geophysical Research, 108(A1), 1011. 10.1029/2002JA009323

[jgra57717-bib-0041] Hwang, K.‐J. , Sibeck, D. G. , Choi, E. , Chen, L. , Ergun, R. E. , Khotyaintsev, Y. , et al. (2017). Magnetospheric Multiscale mission observations of the outer electron diffusion region. Geophysical Research Letters, 44(5), 2049–2059. 10.1002/2017GL072830

[jgra57717-bib-0042] Kacem, I. , Jacquey, C. , Genot, V. , Lavraud, B. , Vernisse, Y. , Marchaudon, A. , et al. (2018). Magnetic reconnection at a thin current sheet separating two interlaced flux tubes at the Earth’s magnetopause. Journal of Geophysical Research: Space Physics, 123(3), 1779–1793. 10.1002/2017JA024537

[jgra57717-bib-0043] Khotyaintsev, Y. V. , Graham, D. B. , Norgren, C. , & Vaivads, A. (2019). Collisionless magnetic reconnection and waves: Progress review. Frontiers in Astronomy and Space Sciences, 6, 70. 10.3389/fspas.2019.00070

[jgra57717-bib-0044] Khotyaintsev, Y. V. , Graham, D. B. , Steinvall, K. , Alm, L. , Vaivads, A. , Johlander, A. , et al. (2020). Electron heating by Debye‐scale turbulence in guide‐field reconnection. Physical Review Letters, 124(4), 045101. 10.1103/PhysRevLett.124.045101 32058767

[jgra57717-bib-0045] Khrabrov, A. V. , & Sonnerup, B. U. Ö. (1998). DeHoffmann‐Teller analysis. In G. Paschmann , & P. W. Daly (Eds.), Analysis methods for multi‐spacecraft data (pp. 221–248). Int. Space Sci. Inst., Bern, Switzerland, and Eur. Space Agency (chap. 9).

[jgra57717-bib-0046] Lindqvist, P.‐A. , Olsson, G. , Torbert, R. B. , King, B. , Granoff, M. , Rau, D. , et al. (2016). The Spin‐plane Double Probe electric field instrument for MMS. Space Science Reviews, 199(1–4), 137–165. 10.1007/s11214-014-0116-9

[jgra57717-bib-0047] Liu, Y.‐H. , Hesse, M. , Guo, F. , Daughton, W. , Li, H. , Cassak, P. A. , & Shay, M. A. (2017). Why does steady‐state magnetic reconnection have a maximum local rate of order 0.1? Physical Review Letters, 118(8), 085101. 10.1103/PhysRevLett.118.085101 28282209

[jgra57717-bib-0048] Marshall, A. T. , Burch, J. L. , Reiff, P. H. , Webster, J. M. , Denton, R. E. , Rastaetter, L. , et al. (2022). Lower hybrid drift wave motion at a dayside magnetopause X‐line with energy conversion dominated by a parallel electric field. Physics of Plasmas, 29(1), 012905. 10.1063/5.0071159

[jgra57717-bib-0049] Nakamura, T. K. M. , Hasegawa, H. , Genestreti, K. J. , Denton, R. E. , Phan, T. D. , Stawarz, J. E. , et al. (2021). Fast cross‐scale energy transfer during turbulent magnetic reconnection. Geophysical Research Letters, 48(13), e2021GL093524. 10.1029/2021GL093524

[jgra57717-bib-0050] Nakamura, T. K. M. , Nakamura, R. , Narita, Y. , Baumjohann, W. , & Daughton, W. (2016). Multi‐scale structures of turbulent magnetic reconnection. Physics of Plasmas, 23(5), 052116. 10.1063/1.4951025

[jgra57717-bib-0051] Nishida, A. (1989). Can random reconnection on the magnetopause produce the low latitude boundary layer? Geophysical Research Letters, 16(3), 227–230. 10.1029/gl016i003p00227

[jgra57717-bib-0052] Øieroset, M. , Phan, T. D. , Drake, J. F. , Eastwood, J. P. , Fuselier, S. A. , Strangeway, R. J. , et al. (2019). Reconnection with magnetic flux pileup at the interface of converging jets at the magnetopause. Geophysical Research Letters, 46(4), 1937–1946. 10.1029/2018GL08094

[jgra57717-bib-0053] Øieroset, M. , Phan, T. D. , Eastwood, J. P. , Fujimoto, M. , Daughton, W. , Shay, M. A. , et al. (2011). Direct evidence for a three‐dimensional magnetic flux rope flanked by two active magnetic reconnection X lines at Earth’s magnetopause. Physical Review Letters, 107(16), 165007. 10.1103/PhysRevLett.107.165007 22107399

[jgra57717-bib-0054] Øieroset, M. , Phan, T. D. , Haggerty, C. , Shay, M. A. , Eastwood, J. P. , Gershman, D. J. , et al. (2016). MMS observations of large guide field symmetric reconnection between colliding reconnection jets at the center of a magnetic flux rope at the magnetopause. Geophysical Research Letters, 43(11), 5536–5544. 10.1002/2016GL069166

[jgra57717-bib-0055] Paschmann, G. , & Sonnerup, B. U. Ö. (2008). Proper frame determination and Walén test. In G. Paschmann , & P. W. Daly (Eds.), Multi‐spacecraft analysis methods revisited. ISSI SR‐008 (pp. 65–74). ESA Publications Division.

[jgra57717-bib-0056] Phan, T. D. , Dunlop, M. W. , Paschmann, G. , Klecker, B. , Bosqued, J. M. , Reme, H. , et al. (2004). Cluster observations of continuous reconnection at the magnetopause under steady interplanetary magnetic field conditions. Annales Geophysicae, 22(7), 2355–2367. 10.5194/angeo-22-2355-2004

[jgra57717-bib-0057] Pollock, C. , Moore, T. , Jacques, A. , Burch, J. , Gliese, U. , Saito, Y. , et al. (2016). Fast plasma investigation for Magnetospheric Multiscale. Space Science Reviews, 199(1–4), 331–406. 10.1007/s11214-016-0245-4

[jgra57717-bib-0058] Pritchett, P. L. (2013). The influence of intense electric fields on three‐dimensional asymmetric magnetic reconnection. Physics of Plasmas, 20(6), 061204. 10.1063/1.4811123

[jgra57717-bib-0059] Pritchett, P. L. , & Coroniti, F. V. (2004). Three‐dimensional collisionless magnetic reconnection in the presence of a guide field. Journal of Geophysical Research, 109(A1), A01220. 10.1029/2003JA009999

[jgra57717-bib-0060] Pu, Z. Y. , Raeder, J. , Zhong, J. , Bogdanova, Y. V. , Dunlop, M. , Xiao, C. J. , et al. (2013). Magnetic topologies of an in vivo FTE observed by Double Star/TC‐1 at Earth’s magnetopause. Geophysical Research Letters, 40(14), 3502–3506. 10.1002/grl.50714

[jgra57717-bib-0061] Qi, Y. , Russell, C. T. , Jia, Y.‐D. , & Hubbert, M. (2020). Temporal evolution of flux tube entanglement at the magnetopause as observed by the MMS satellites. Geophysical Research Letters, 47(23), e2020GL090314. 10.1029/2020GL090314

[jgra57717-bib-0062] Raeder, J. (2006). Flux transfer events: 1. Generation mechanism for strong southward IMF. Annales Geophysicae, 24, 381–392. 10.5194/angeo-24-381-2006

[jgra57717-bib-0063] Rager, A. C. , Dorelli, J. C. , Gershman, D. J. , Uritsky, V. , Avanov, L. A. , Torbert, R. B. , et al. (2018). Electron crescent distributions as a manifestation of diamagnetic drift in an electron‐scale current sheet: Magnetospheric Multiscale observations using new 7.5 ms Fast Plasma Investigation moments. Geophysical Research Letters, 45(2), 578–584. 10.1002/2017GL076260 29576666 PMC5856066

[jgra57717-bib-0064] Russell, C. T. , Anderson, B. J. , Baumjohann, W. , Bromund, K. R. , Dearborn, D. , Fischer, D. , et al. (2016). The Magnetospheric Multiscale magnetometers. Space Science Reviews, 199(1–4), 189–256. 10.1007/s11214-014-0057-3

[jgra57717-bib-0065] Schindler, K. , Hesse, M. , & Birn, J. (1988). General magnetic reconnection, parallel electric fields, and helicity. Journal of Geophysical Research, 93(A6), 5547–5557. 10.1029/JA093iA06p05547

[jgra57717-bib-0066] Shi, Q. Q. , Shen, C. , Dunlop, M. W. , Pu, Z. Y. , Zong, Q.‐G. , Liu, Z.‐X. , et al. (2006). Motion of observed structures calculated from multi‐point magnetic field measurements: Application to Cluster. Geophysical Research Letters, 33(8), L08109. 10.1029/2005GL025073

[jgra57717-bib-0067] Shi, Q. Q. , Shen, C. , Pu, Z. Y. , Dunlop, M. W. , Zong, Q.‐G. , Zhang, H. , et al. (2005). Dimensional analysis of observed structures using multipoint magnetic field measurements: Application to Cluster. Geophysical Research Letters, 32(12), L12105. 10.1029/2005GL022454

[jgra57717-bib-0068] Shi, Q. Q. , Tian, A. M. , Bai, S. C. , Hasegawa, H. , Degeling, A. W. , Pu, Z. Y. , et al. (2019). Dimensionality, coordinate system and reference frame for analysis of in‐situ space plasma and field data. Space Science Reviews, 215(4), 35. 10.1007/s11214-019-0601-2

[jgra57717-bib-0069] Sonnerup, B. U. Ö. , Hasegawa, H. , Denton, R. E. , & Nakamura, T. K. M. (2016). Reconstruction of the electron diffusion region. Journal of Geophysical Research: Space Physics, 121(5), 4279–4290. 10.1002/2016JA022430 PMC9286637 35864949

[jgra57717-bib-0070] Sonnerup, B. U. Ö. , Hasegawa, H. , Teh, W.‐L. , & Hau, L.‐N. (2006). Grad‐Shafranov reconstruction: An overview. Journal of Geophysical Research, 111(A9), A09204. 10.1029/2006JA011717

[jgra57717-bib-0071] Stawarz, J. E. , Eastwood, J. P. , Genestreti, K. J. , Nakamura, R. , Ergun, R. E. , Burgess, D. , et al. (2018). Intense electric fields and electron‐scale substructure within magnetotail flux ropes as revealed by the Magnetospheric Multiscale mission. Geophysical Research Letters, 45(17), 8783–8792. 10.1029/2018GL079095

[jgra57717-bib-0072] Steinvall, K. , Khotyaintsev, Y. V. , Graham, D. B. , Vaivads, A. , Le Contel, O. , & Russell, C. T. (2019). Observations of electromagnetic electron holes and evidence of Cherenkov whistler emission. Physical Review Letters, 123(25), 255101. 10.1103/PhysRevLett.123.255101 31922784

[jgra57717-bib-0073] Torbert, R. B. , Dors, I. , Argall, M. R. , Genestreti, K. J. , Burch, J. L. , Farrugia, C. J. , et al. (2020). A new method of 3‐D magnetic field reconstruction. Geophysical Research Letters, 47(3), e2019GL085542. 10.1029/2019GL085542

[jgra57717-bib-0074] Wang, S. , Bessho, N. , Graham, D. B. , Le Contel, O. , Wilder, F. D. , Khotyaintsev, Y. V. , et al. (2022). Whistler waves associated with electron beams in magnetopause reconnection diffusion regions. Journal of Geophysical Research: Space Physics, 127(9), e2022JA030882. 10.1029/2022JA030882

[jgra57717-bib-0075] Webster, J. M. , Burch, J. L. , Reiff, P. H. , Daou, A. G. , Genestreti, K. J. , Graham, D. B. , et al. (2018). Magnetospheric Multiscale dayside reconnection electron diffusion region events. Journal of Geophysical Research: Space Physics, 123(6), 4858–4878. 10.1029/2018JA025245

[jgra57717-bib-0076] Wetherton, B. A. , Egedal, J. , Le, A. , & Daughton, W. (2022). Generation of a strong parallel electric field and embedded electron jet in the exhaust of moderate guide field reconnection. Geophysical Research Letters, 49(14), e2022GL098907. 10.1029/2022GL098907

[jgra57717-bib-0077] Wilder, F. D. , Ergun, R. E. , Hoilijoki, S. , Webster, J. , Argall, M. R. , Ahmadi, N. , et al. (2019). A survey of plasma waves appearing near dayside magnetopause electron diffusion region events. Journal of Geophysical Research: Space Physics, 124(10), 7837–7849. 10.1029/2019JA027060

[jgra57717-bib-0078] Wilder, F. D. , Ergun, R. E. , Newman, D. L. , Goodrich, K. A. , Trattner, K. J. , Goldman, M. V. , et al. (2017). The nonlinear behavior of whistler waves at the reconnecting dayside magnetopause as observed by the Magnetospheric Multiscale mission: A case study. Journal of Geophysical Research: Space Physics, 122(5), 5487–5501. 10.1002/2017JA024062

[jgra57717-bib-0079] Zenitani, S. , Hesse, M. , Klimas, A. , & Kuznetsova, M. (2011). New measure of the dissipation region in collisionless magnetic reconnection. Physical Review Letters, 106(19), 195003. 10.1103/PhysRevLett.106.195003 21668168

[jgra57717-bib-0081] Zhong, J. , Pu, Z. Y. , Dunlop, M. W. , Bogdanova, Y. V. , Wang, X. G. , Xiao, C. J. , et al. (2013). Three‐dimensional magnetic flux rope structure formed by multiple sequential X‐line reconnection at the magnetopause. Journal of Geophysical Research: Space Physics, 118(5), 1904–1911. 10.1002/jgra.50281

[jgra57717-bib-0080] Zhong, Z. H. , Graham, D. B. , Khotyaintsev, Y. V. , Zhou, M. , Le Contel, O. , Tang, R. X. , & Deng, X. H. (2021). Whistler and broadband electrostatic waves in the multiple X‐line reconnection at the magnetopause. Geophysical Research Letters, 48(4), e2020GL091320. 10.1029/2020GL091320

[jgra57717-bib-0082] Zhong, Z. H. , Zhou, M. , Deng, X. H. , Song, L. J. , Graham, D. B. , Tang, R. X. , et al. (2021). Three‐dimensional electron‐scale magnetic reconnection in Earth’s magnetosphere. Geophysical Research Letters, 48(1), e2020GL090946. 10.1029/2020GL090946

