# Supplementary material for: Ion‐Scale Magnetic Flux Rope Generated From Electron‐Scale Magnetopause Current Sheet: Magnetospheric Multiscale Observations
Source: J Geophys Res Space Phys. 2023 Mar 24;128(3):e2022JA031092. doi: 10.1029/2022JA031092 (PMC10909477; doi:10.1029/2022JA031092)
Supplement: Supplementary file 1 — Supporting Information S1 [file JGRA-128-e2022JA031092-s001.docx]

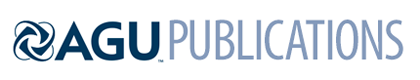


Journal of Geophysical Research: Space Physics

Supporting Information for

**Ion-Scale Magnetic Flux Rope Generated from Electron-Scale Magnetopause Current Sheet: Magnetospheric Multiscale Observations**

H. Hasegawa^1,2^, R. E. Denton^3^, K. Dokgo^2^, K.-J. Hwang^2^, T. K. M. Nakamura^4^, and J. L. Burch^2^

^1^Institute of Space and Astronautical Science, Japan Aerospace Exploration Agency, Sagamihara, Japan.

^2^Southwest Research Institute, San Antonio, TX, USA.

^3^Department of Physics and Astronomy, Dartmouth College, Hanover, NH, USA.

^4^Space Research Institute, Austrian Academy of Sciences, Graz, Austria.

**Contents of this file**

Figures S1 to S5

Table S1

**Introduction**

The supporting information includes figures supporting our interpretations of Magnetospheric Multiscale (MMS) observations of an ion-scale magnetic flux rope in an electron-scale magnetopause current sheet on 8 December 2015 at 11:20:43 UT.


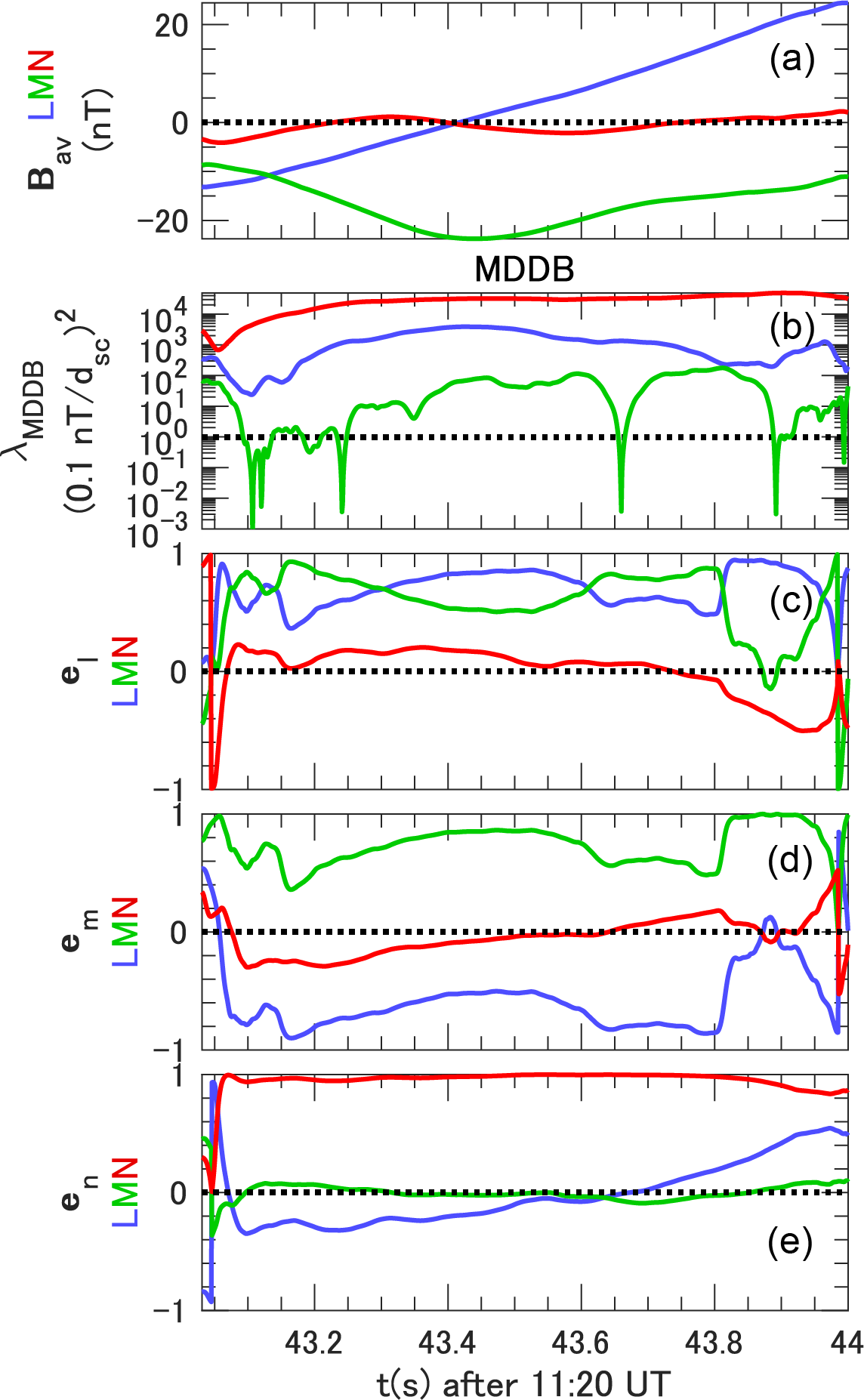


Figure S1. Results from the Maximum Directional Derivative method (Shi et al., 2019) applied to magnetic field data (MDDB) taken by the four MMS spacecraft for interval 11:20:43-11:20:44 UT. (a) Four-spacecraft and 0.3 s running average of the measured magnetic field in LMN coordinates estimated by a hybrid method (Denton et al., 2018). GSM components of the LMN axes are: $\mathbf{L}_{\mathbf{h}}\mathbf{=}\left( \mathbf{0.3964, -0.0956, 0.9131} \right)$, $\mathbf{M}_{\mathbf{h}}\mathbf{=}\left( \mathbf{0.0361, -0.9922, -0.1196} \right)$, and $\mathbf{N}_{\mathbf{h}}\mathbf{=}\left( \mathbf{0.9174, 0.0803, -0.3898} \right)$. (b) Eigenvalues from the MDDB method, whose ratio can be used as a measure of the structure dimensionality. The spacecraft separation is $\mathbf{d}_{\mathbf{sc}}\mathbf{=15.2}$ km. (c-e) Instantaneous orientations in LMN of the intermediate, minimum, and maximum gradient directions ($\mathbf{e}_{\mathbf{l}}$, $\mathbf{e}_{\mathbf{m}}$, and $\mathbf{e}_{\mathbf{n}}$, respectively) from MDDB.


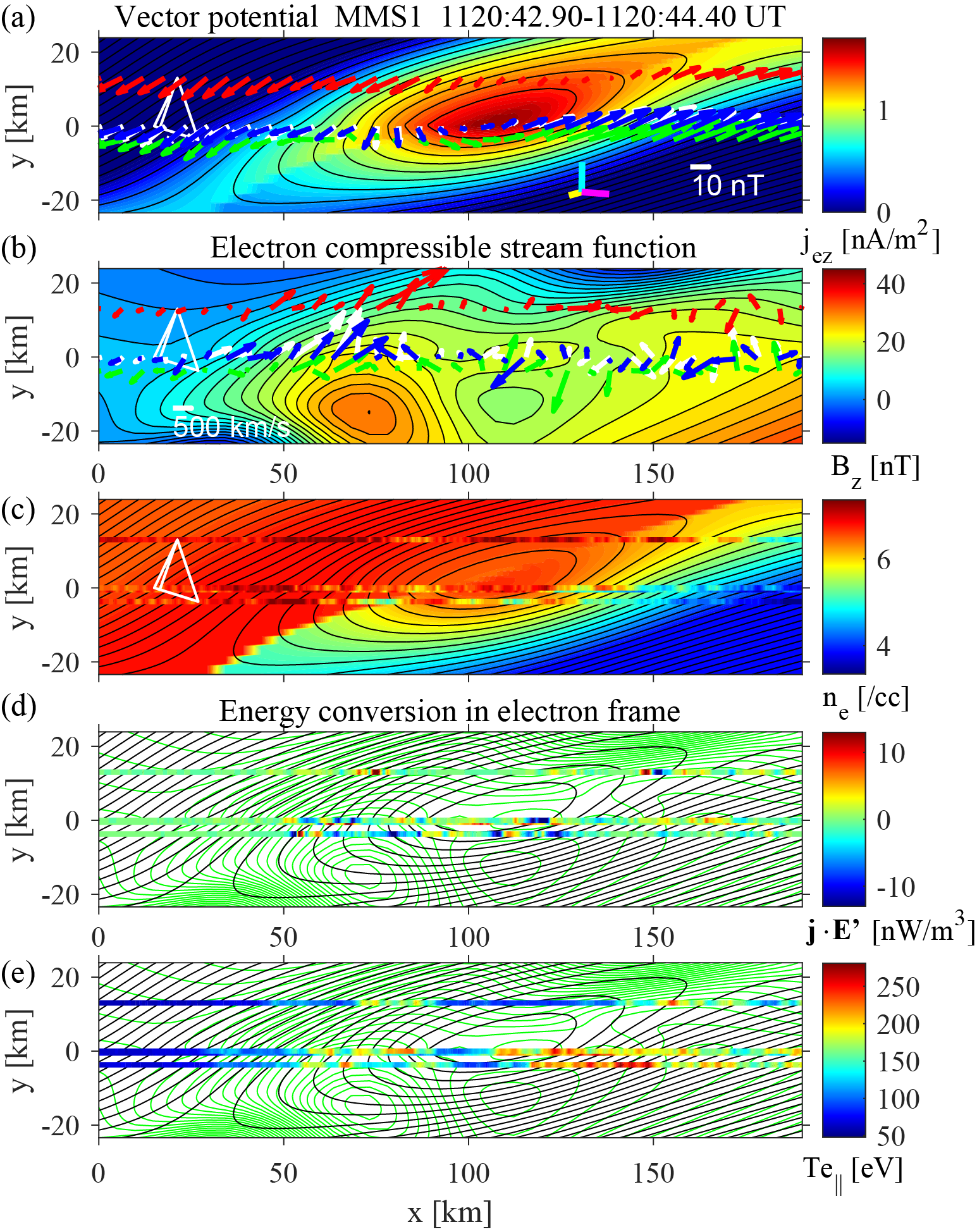


Figure S2. Field maps from the EMHD reconstruction applied to MMS1 data with 7.5 ms resolution, in the same format as Figure 4. In Figures S2c-S2e, the MMS1 paths colored by the measured electron density, energy conversion rate, and parallel electron temperature, respectively, are nearly on top of the MMS4 paths.


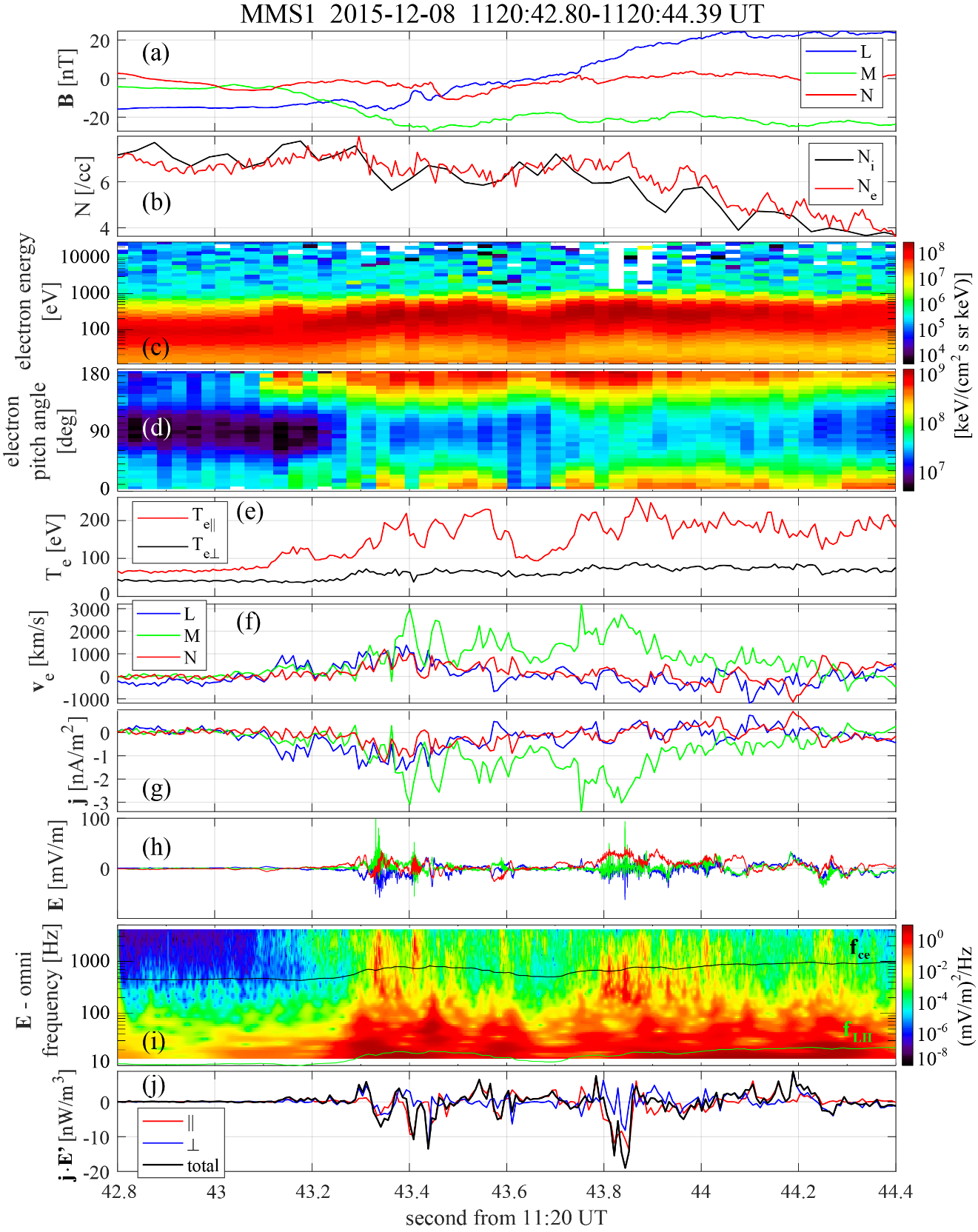


Figure S3. Burst-mode MMS1 data around the reconstructed ion-scale magnetic flux rope encountered at 11:20:43 UT, in the same format as Figures 8a-8j.


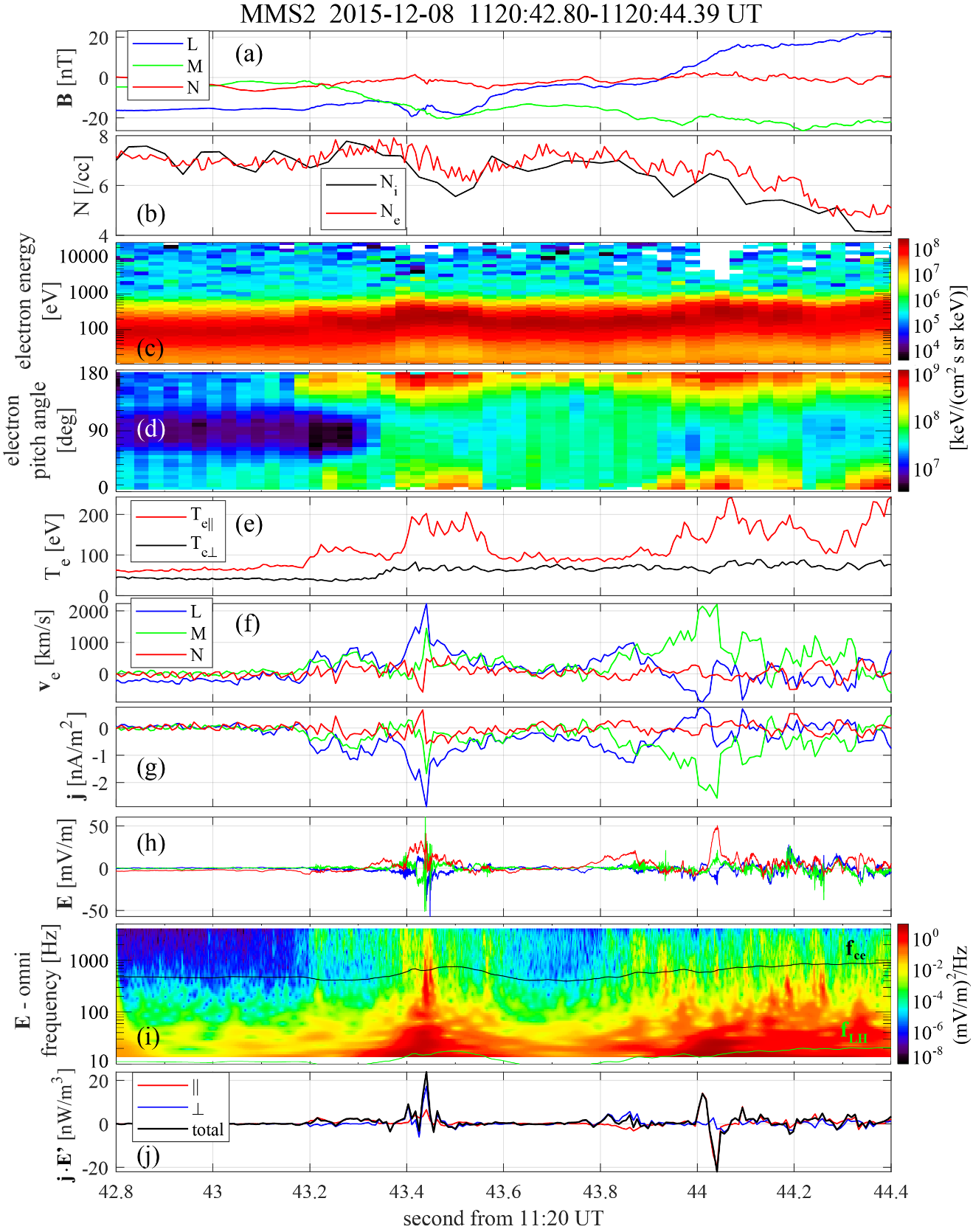


Figure S4. Burst-mode MMS2 data around the reconstructed ion-scale magnetic flux rope encountered at 11:20:43 UT, in the same format as Figures 8a-8j.


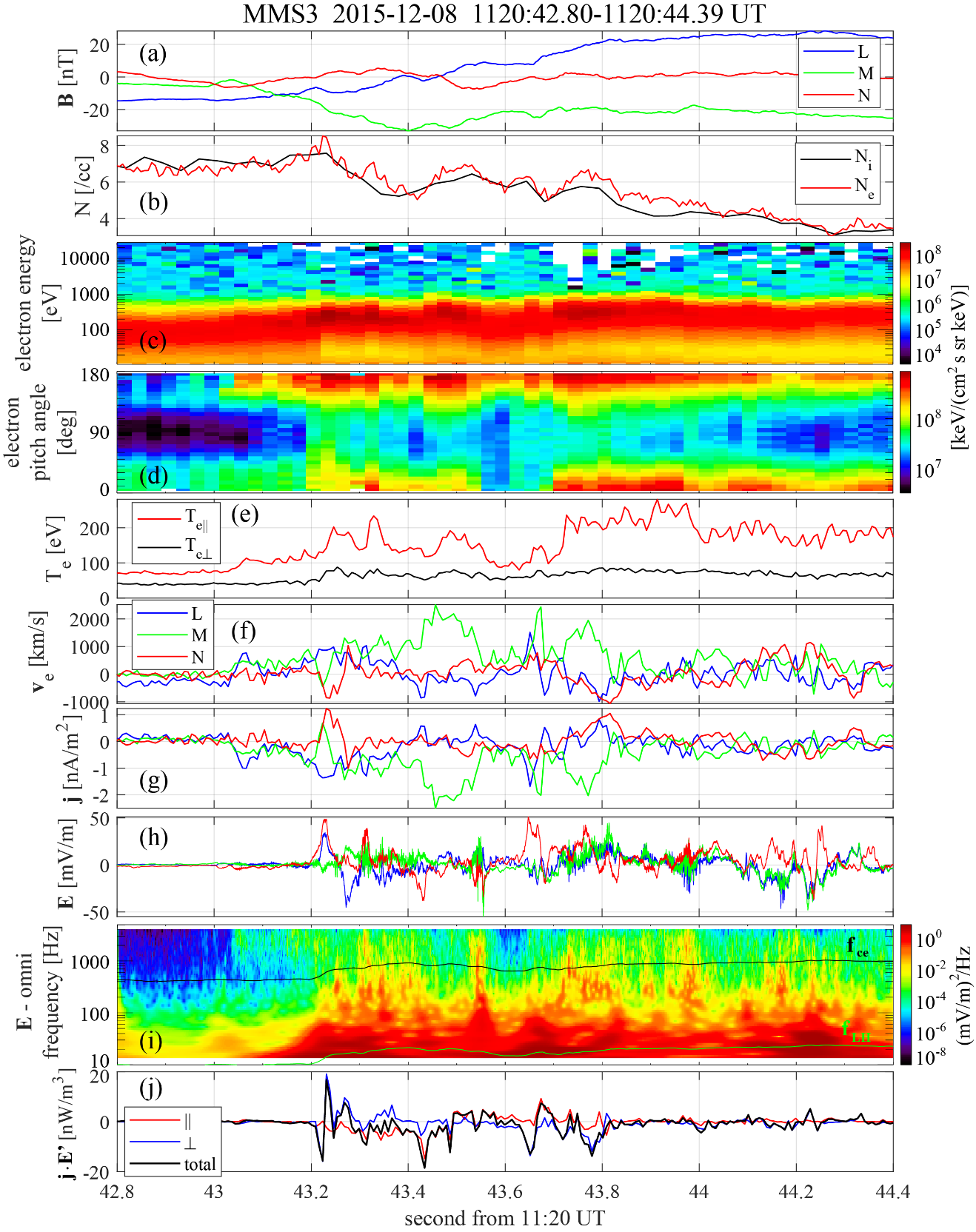


Figure S5. Burst-mode MMS3 data around the reconstructed ion-scale magnetic flux rope encountered at 11:20:43 UT, in the same format as Figures 8a-8j.

Table S1. Results from the EMHD reconstruction for all four MMS spacecraft in the order of electron-scale current sheet crossing.

|  | MMS3 | MMS4 | MMS1 | MMS2 |
| --- | --- | --- | --- | --- |
| Interval (UT) | 1120:42.6-1120:44.1 | 1120:42.9-1120:44.4 | 1120:42.9-1120:44.4 | 1120:43.1-1120:44.6 |
| $z_{\mathrm{EMHD}}$ relative to MMS4 (km) | 7.1 | 0.0 | 14.2 | 7.5 |
| ${\mathrm{cc}_{B}}^{a}$ | 0.9309 | 0.9755 | 0.9679 | 0.9176 |
| ${\mathrm{cc}_{V}}^{b}$ | 0.5461 | 0.5924 | 0.5743 | 0.3167 |

^a^$\mathrm{cc}_{B}$: correlation coefficient between the predicted and measured magnetic field components, as shown in Figure 5a.

^b^$\mathrm{cc}_{V}$: correlation coefficient between the predicted and measured electron velocity components in the structure-rest frame, as shown in Figure 5b.
